# Crosstalk between macrophage-derived PGE_2_ and tumor UHRF1 drives hepatocellular carcinoma progression

**DOI:** 10.7150/thno.69494

**Published:** 2022-05-01

**Authors:** Jian Zhang, Hongyan Zhang, Xiuli Ding, Jia Hu, Yongkui Li, Jinxiang Zhang, Hui Wang, Shanshan Qi, Aqing Xie, Jie Shi, Mengxi Xiang, Yawen Bin, Guobin Wang, Lin Wang, Zheng Wang

**Affiliations:** 1 Department of Clinical Laboratory, Union Hospital, Tongji Medical College, Huazhong University of Science and Technology, Wuhan, Hubei, China 430022.; 2 Research Center for Tissue Engineering and Regenerative Medicine, Union Hospital, Tongji Medical College, Huazhong University of Science and Technology, Wuhan, Hubei, China 430022.; 3 Department of Surgery, Union Hospital, Tongji Medical College, Huazhong University of Science and Technology, Wuhan, Hubei, China 430022.; 4 Department of Human Genetics, Tongji Medical College, Huazhong University of Science and Technology, Wuhan, Hubei, China 430030.; 5 Department of Gastrointestinal Surgery, Union Hospital, Tongji Medical College, Huazhong University of Science and Technology, Wuhan, Hubei, China 430022.

**Keywords:** hepatocellular carcinoma, TAMs, UHRF1, epigenetic regulator, crosstalk, miRNA

## Abstract

**Background:** Tumor-associated macrophages (TAMs) and dysregulated tumor epigenetics contribute to hepatocellular carcinoma (HCC) progression. However, the mechanistic interactions between TAMs and tumor epigenetics remain poorly understood.

**Methods:** Immunohistochemistry and multiplexed fluorescence staining were performed to evaluate the correlation between TAMs numbers and UHRF1 expression in human HCC tissues. PGE_2_ neutralizing antibody and COX-2 inhibitor were used to analyze the regulation of TAMs isolated from HCC tissues on UHRF1 expression. Multiple microRNA prediction programs were employed to identify microRNAs that target *UHRF1* 3'UTR. Luciferase reporter assay was applied to evaluate the regulation of miR-520d on UHRF1 expression. Chromatin immunoprecipitation (ChIP) assays were performed to assess the abundance of H3K9me2 in the *KLF6* promoter and DNMT1 in the *CSF1* promoter regulated by UHRF1. The functional roles of TAM-mediated oncogenic network in HCC progression were verified by *in vitro* colony formation assays, *in vivo* xenograft experiments and analysis of clinical samples.

**Results:** Here, we find that TAMs induce and maintain high levels of HCC UHRF1, an oncogenic epigenetic regulator. Mechanistically, TAM-derived PGE_2_ stimulates UHRF1 expression by repressing miR-520d that targets the 3'-UTR of *UHRF1* mRNA. In consequence, upregulated UHRF1 methylates H3K9 to diminish tumor KLF6 expression, a tumor inhibitory transcriptional factor that directly transcribes miR-520d. PGE_2_ reduces KLF6 occupancy in the promoter of miR-520d, dampens miR-520d expression, and sustains robust UHRF1 expression. Moreover, UHRF1 promotes CSF1 expression by inducing DNA hypomethylation of the *CSF1* promoter and supports TAM accumulation.

**Conclusions:** Capitalizing on studies on HCC cells and tissues, animal models, and clinical information, we reveal a previously unappreciated TAM-mediated oncogenic network via multiple reciprocal enforcing molecular nodes. Targeting this network may be an approach to treat HCC patients.

## Introduction

Hepatocellular carcinoma (HCC) has high morbidity and mortality worldwide 1. Inflammation 2 and dysregulated tumor epigenetic changes 3 contribute to cancer initiation, progression, and dissemination. Tumor-associated macrophages (TAMs) are major inflammatory cells in the tumor microenvironment. TAMs can target the immune system to suppress anti-tumor immunity, promote tumor angiogenesis and cancer invasiveness, and affect tumor therapy 4-6. TAMs may be associated with tumor epigenetic alterations, including DNA methylation and histone modifications 7, 8. However, the mechanistic interplay between TAMs and the tumor epigenetic regulatory network remains unclear in HCC.

As a critical oncogenic epigenetic regulator, the ubiquitin-like with PHD and ring finger domains 1 (UHRF1), orchestrates DNA methylation and histone modifications across genome 9. It has been reported that UHRF1 silences tumor suppressor genes through recruiting epigenetic enzymes, including DNA methyltransferase (DNMT1) and histone lysine methyltransferases (G9a and Suv39H1) 10-13. *UHRF1*, thought to be an oncogene 14, is highly expressed in HCC and can promote HCC initiation and growth in zebrafish 14. However, it is poorly understood how UHRF1 expression is regulated. Although an increasing number of investigations have begun to decode functional roles of UHRF1 in cancer biology 15, 16, few studies are focused on tumor UHRF1's interactions with immune cells, such as TAMs. Whether and how UHRF1 expression and function are connected to TAMs in the HCC microenvironment remains unexplored.

In this work, we answer these questions by showing that TAM-derived PGE_2_ controls tumor UHRF1 expression. We demonstrate that the interaction between macrophages and HCC initiates and sustains an oncogenic feedback loop via the PGE_2_-miR-520d-KLF6-UHRF1-CSF1 network. This network coordinates and sustains HCC progression.

## Materials and Methods

### HCC patients and HCC tissues

Patients diagnosed with HCC were enrolled in this study. All human samples in this study were used with the approval of the local Institutional Review Board, Tongji Medical College, Huazhong University of Science and Technology (Wuhan, China). Tissue microarrays (TMA) consisting of 53 HCC tissues and their paired non-tumor normal tissues (Group 1; paraffin tissues) were obtained from the First Affiliated Hospital of Sun Yat-Sen University (Guangzhou, China). Fresh HCC tissue and their paired adjacent liver tissues (snap-frozen tissues) were from 18 HCC patients who have undergone liver cancer resection at Union Hospital of Huazhong University of Science and Technology (Wuhan, China). These tissues were used for qRT-PCR analysis and DNA methylation analysis. Written consent was obtained from all patients. Relevant gene expression and survival analyses were conducted for HCC patients from The Cancer Genome Atlas (TCGA) database (https://gdc.cancer.gov/), and three HCC datasets from GEO (https://www.ncbi.nlm.nih.gov/geo/) (GSE20596 for microRNAs, GSE10694 for survival, and GSE6764 for correlation analyses).

### Cell lines

Human HCC cell lines (HepG2 and Huh7), immortalized human normal liver cell line (L02), and human embryonic kidney 293 cell line (HEK293T) were purchased from the Institute of Biochemistry and Cell Biology, Chinese Academy of Sciences (Shanghai, China). Murine hepatocellular carcinoma cell line (H22) was purchased from Mito-bio Co. Ltd (Shanghai, China). These cell lines were routinely maintained.

### Animals

Six- to eight-week-old male T-cell-deficient nude mice and BALB/c mice were purchased from Beijing HFK BioTechnology Co. Ltd (Beijing, China). Six- to eight-week-old female NOD-*Prkdc^em26Cd52^II2rg^em26Cd22^*/Nju (NCG) mice were purchased from the Nanjing University Model Animal Institute (Nanjing, China). All studies were performed in accordance with the guidelines of Health Guide for the Care and Use of Laboratory Animals of the National Institutes of Health. All experiments were approved by the Ethics Committee of Union Hospital, Tongji Medical College, Huazhong University of Science and Technology (Wuhan, China).

### Immunohistochemistry and multiplexed fluorescence staining

The tissue microarrays (TMA) consisting of 53 HCC tissues and their paired non-tumor normal tissues were stained for expression of UHRF1 (sc-373750, Santa Cruz biotechnology, USA) and CD68 (M 0876, DAKO, USA). Briefly, after baking in a thermostat dryer at 60 °C for an hour, TMA sections were deparaffinized with xylene and rehydrated. 3% (vol/vol) hydrogen peroxide was used to quench endogenous peroxidase activity for 10 minutes, followed by four 3-minute washes with double-distilled water. Subsequently, the slides were immersed in 0.1 mol/L Tris-HCl solution (pH 9.2) and heated in a microwave oven for 30 minutes. After four 3-minute washes with PBS and being pretreated with PBS containing 5% (wt/vol) bovine serum albumin for 30 minutes, the sections were incubated in a humidified box at 4 °C overnight with a primary antibody. After four 5-minute washes with PBS, the sections were incubated with a biotinylated second antibody (Santa Cruz biotechnology, USA) for 30 minutes at 37 °C, followed by another four 5-minute washes with PBS. The reaction products were visualized using diaminobenzidine (DAKO, USA) for 2 minutes, and counterstained with hematoxylin for 1 to 6 minutes. Images were acquired under a light microscope with a 40× objective lens (Olympus, Japan). UHRF1 expression in TMA sections were scored manually using the H-score method that integrated percentages of positive cells and staining intensities into the formula: (0 × % negative) + (1 × % weak) + (2 × % moderate) + (3 × % strong) 17-19. CD68^+^ cells were counted with averaging positive cell numbers from six randomly selected fields from each tissue spot (n = 6 fields per spot).

Multiplexed fluorescence staining was performed as previously described 20. Tissues were stained with DAPI (C0060, Solarbio, Beijing, China) and CD68 antibody (M 0876, DAKO, USA) plus a CY3-conjugated goat anti-rabbit antibody. Images were acquired under a light microscope with a 40x objective lens (Olympus, Japan). CD68^+^ cells were counted by averaging positive cell numbers from six randomly selected fields from each slide (n = 6 slides per group).

### Tumor formation and macrophage depletion

For tumor formation assays, 10^6^ H22 cells (or H22 cells stably expressing short hairpin RNA against Uhrf1 (shUhrf1) or shRNA nonsense control (shNC)) were subcutaneously injected into BALB/c mice. HepG2 cells (5 × 10^6^) expressing miR-520d, miR-520d mutants, and control vectors were subcutaneously inoculated into the two posterior flanks of the same NCG mice. HepG2 cells (5 × 10^6^) and HepG2 cells stably expressing miR-520d were also subcutaneously injected into different nude mice for tumor growth, tumor formation rates, and mouse survival analysis. For macrophage depletion, 200 µL of Clodronate liposomes (Formumax Scientific Inc., USA) was intraperitoneally injected into mice 2 days before tumor inoculation, followed by intraperitoneal injection of 100 µL of Clodronate liposomes every three days, three times consecutively. Tumor size was measured every 2 days with a Vernier caliper. Tumor formation rates and mouse survival were recorded. The tumors were surgically resected at day 21 post inoculation.

### Celecoxib treatment

10^6^ H22 cells were inoculated subcutaneously into the right flank of each mouse. After the tumor reached 50 mm^3^ following tumor cell injection, the mice were randomized into two groups with eight mice in each group. The control group were intraperitoneally injected with saline. The celecoxib-treatment group received gavage of celecoxib at 150 mg/kg once a day. Tumor size was measured every 2 days with a Vernier caliper and tumor volume was calculated using the formula: (V = W^2^ × L/ 2), where W and L were the perpendicular smaller and large diameters, respectively. At the endpoint, the tumors were resected after euthanasia of animals. The tumors were immediately soaked into 4% paraformaldehyde for immunohistochemistry (IHC) staining.

### TAM/macrophage isolation and culture

TAMs were isolated from fresh HCC samples from five HCC patients at Union Hospital (Wuhan, China). Briefly, the tissues were cut into 1 to 2 mm^3^ pieces and digested at 37 °C for 2 hours with 5 mL of DMEM medium containing 10% FBS, 2 mg/mL hyaluronidase, and 2 mg/mL collagenase I. First, the cell suspension was filtered through a mesh (500-μm pore size, BD Biosciences, USA) and filtered once more with a cell strainer (70-μm pore size, BD Biosciences, USA). Then, 3 mL of cell suspension was added on the top of 4 mL of 75% Ficoll in the middle of a 15 mL tube with 3 mL of 100% Ficoll at the bottom. The tube was centrifuged at 2,000 rpm for 30 minutes. The cell layer in the interphase between 75% and 100% Ficoll was collected. CD14^+^ macrophages were isolated by a magnetic-activated cell sorting using a CD14 Isolation Kit (Miltenyi Biotec, Germany) according to the manufacturers' instructions. Monocytes (normal macrophages) were isolated from peripheral blood of healthy volunteers as previously described 20*.* These macrophages or TAMs were cultured with 10% FBS RPMI 1640 medium. For supernatant collection, after being pretreated with celecoxib or control medium for 12 hours, macrophages from healthy volunteers or TAMs were washed by PBS and cultured with fresh medium for another 24 hours. The medium (i.e. supernatant) was then collected. HepG2 cells in the lower chamber were co-cultured with TAMs or macrophages in the upper chamber in a transwell culture system.

### Antibody neutralization

For PGE_2_ neutralization, TAMs (10^5^) isolated from human HCC patients were cultured in a well of a 6-well plate with 2 mL RPMI medium for 24 hours. The supernatants were collected and mixed with 2 μg/mL PGE_2_ neutralizing antibody (anti-PGE_2_, 360150, Cayman Chemical, USA) or isotype IgG for 24 hours. HepG2 or Huh7 cells were incubated with the supernatants for 24 hours before analysis. For neutralization of CSF1 and CCL14, the supernatants were harvested from 10^7^ HepG2 cells stably expressing UHRF1 (or transfected with control vector) that were cultured in 10-cm dishes for 48 hours. The supernatants were mixed with 2 μg/mL of CSF1 antibody (AF216, R&D Systems, USA), CCL14 antibody (MAB3241, R&D Systems, USA) or isotype IgG for 24 hours. These supernatants were used to treat TAMs for 24 or 48 hours before analysis.

### Cell transfection

For RNA (siRNA, miRNA or miRNA inhibitor) transfection, HepG2 or HEK293 cells were seeded onto 6-well plates (2 mL culture medium per well) and grew up to 70% confluency. The cells were transfected with 100 nM RNA using Lipofectamine 2000 (Invitrogen, Carlsbad, CA, USA) according to the manufacturers' instructions. For plasmid transient transfection, HepG2 or L02 cells were seeded in 6-well plates. When reaching 70% confluency, the cells were transfected with 2 μg of DNA per well using Lipofectamine 2000 (Invitrogen, Carlsbad, CA, USA). The cells were harvested for analysis 48 hours after transfection. For stable transfection, HepG2 cells stably expressing miR-520d, short hairpin RNA against *UHRF1* (shUHRF1) or short hairpin RNA against *KLF6* (shKLF6), and murine HCC H22 cells stably expressing shUhrf1 were selected with 3 μg/mL puromycin (Sigma-Aldrich, St. Louis, MO, USA). Briefly, shUHRF1 (or shUhrf1 against mouse *Uhrf1* gene) and shKLF6 sequences were constructed into the pLKO.1-Puro plasmid and packaged into Lenti-viral particles in HEK293T cells. The Lentiviral particles were used to infect HepG2 or H22 cells according to the manufacturers' instructions. 10^5^ HepG2 cells were transfected with miR-520d expression plasmid (2 μg) using Lipofectamine 2000 (Invitrogen, Carlsbad, CA, USA) according to the manufacturers' protocols. After 48 hours, cells were selected using 3 μg/mL puromycin (Sigma-Aldrich, St. Louis, MO, USA). The selection medium was replaced every 3 days for 3 weeks. All stably transfected cells were cultured in the medium containing 1.5 μg/mL puromycin. For generating HepG2 cells stably expressing both shUHRF1 and shKLF6, a pSilencer vector containing shKLF6 sequence was transfected into shUHRF1 stably expressing HepG2 cells that were later selected with 400 μg/mL G418 (Sigma-Aldrich, St. Louis, MO, USA). The transfection efficiency of stably transfected cells was confirmed by quantitative real-time PCR or Western blotting. The shRNA and siRNA used were listed in the Table S3 (Supplementary Information).

### CRISPR/Cas9 editing of UHRF1

For CRISPR/Cas9 (clustered regularly interspaced short palindromic repeats associated nuclease Cas9) editing of *UHRF1*, the sgRNA targeting genomic *UHRF1* exon was designed and cloned into Lenti-CRISPR-v2 plasmid (sgRNA targeting *UHRF1*, oligo1: 5'-CACCGAGGTTCGGACCATGGACGGG-3'; oligo2: 5'-AAACCCCGTCCATGGTCCGAACCTC-3'). HepG2 and Huh7 cells were infected with lentiviruses and cultured with puromycin (3 μg/ml for HepG2 cells and 1.5 μg/ml for Huh7 cells) for one week. Then the monoclonal cells were cultured in 96-well plates. Drug-resistant clones were subsequently selected. Genome DNA sequencing, and Western blot were performed to determine whether UHRF1 was knockout (*UHRF1* genome DNA PCR and sequencing primers, Forward: 5'-CAACCCCGACTCCTTAGAGCAT-3'; Reverse: 5'-TTGGTGGTGGATGTTTAAAAAAGAA-3'). UHRF1 homozygous knockout in HepG2 cells could not be generated. Instead, we obtained UHRF1 heterozygous knockout HepG2 cells (Figure S4K, Supplymentary Information). UHRF1 knockout experiments failed in Huh7 cells (data not shown). The possible reason for not being able to obtain homozygous UHRF1 knockout in HepG2 and Huh7 cells might be due to UHRF1's importance to cell survival as suggested by previous study 21, 22.

### RNA extraction and quantitative real-time PCR analysis

Total RNA was obtained from Trizol (Invitrogen)-lysed samples, and 1 mg of total RNA was reversely transcribed into cDNA using M-MLV reverse transcriptase (Thermo Scientific, Hudson, NH, USA). Quantitative real-time PCR was performed in triplicates in a StepOnePlus Real-time PCR system (Applied Biosystems7500, Foster City, USA) with a standard SYBR Green PCR kit (Takara Shuzo Co. Ltd, Kyoto, Japan). The Ct values were calculated using the 2^-△△CT^ method. Human *GAPDH*, human *U6*, and mouse *Hprt* served as endogenous house-keeping genes. The primers used are listed in the Table S2 (Supplementary Information).

### Chromatin immunoprecipitation (ChIP) and re-ChIP assays

ChIP analysis was performed as described previously 20. Briefly, after being crosslinked by 1% formaldehyde for 10 minutes at 37 °C, the cells were resuspended in 300 mL of lysis buffer and sonicated for 10 minutes. The supernatants were incubated with specific antibodies against UHRF1 (sc-373750, Santa Cruz biotechnology, USA), KLF6 (sc-7158, Santa Cruz biotechnology, USA), DNMT1 (sc-271729, Santa Cruz biotechnology, USA), H3K9me2 (A2359, ABclonal, USA), or immunoglobulin G control (Millipore, USA). The immunoprecipitated DNA was then purified using a DNA purification kit (QIAGEN, Germany) and subjected to PCR amplification. For re-ChIP assays, after being combined with protein A agarose beads and the indicated primary antibodies, the complexes were washed and sequentially eluted from the first ChIP by incubation with 10 mM DTT in 1× TE for 30 minutes at 37 °C. The DNA-protein-antibody complexes were then diluted 20 times with dilution buffer and subjected to a second round of immunoprecipitation with the indicated antibodies. After elution and DNA purification, extracted DNA was analyzed by PCR using primers spanning the proximal promoter regions of target genes. The PCR products were normalized to the input. The specific primers are listed in the Table S2 (Supplymentary Information).

### DNA methylation analysis

Bisulfite modification of genomic DNA was carried out using an EZ DNA Methylation-Direct™ Kit (Zymo Research, USA). The DNA methylation levels in the CSF1 promoter in tissue samples were detected using a MethylCollector™ Ultra Kit (Active Motif, Carlsbad, USA). Genomic DNA isolation, bisulfite conversion, and PCR conditions were performed as previously described 23. The primers used for amplification of the miR-520d promoter and the CSF1 promoter are listed in the Table S2 (Supplementary Information). PCR products were analyzed by pyrosequencing to obtain a quantitative dataset for individual CpG sites.

### Western blotting

Protein extracts were probed with antibodies against human UHRF1 (sc-373750, Santa Cruz biotechnology, USA), KLF6 (sc-7158, Santa Cruz biotechnology, USA), COX-2 (A5787, ABclonal, USA), CSF1 (AF216, R&D Systems, USA), DNMT1 (sc-271729, Santa Cruz biotechnology, USA), and CCL14 (MAB3241, R&D Systems, USA) or β-actin (A5441, Sigma, USA). Stained blots were visualized with chemiluminescence assays using ECL detection reagents (Millipore, USA).

### Enzyme-linked immunosorbent assay (ELISA)

The amount of PGE_2_ was detected using ELISA kits (KGE004B, R&D Systems, USA). The serum and the supernatants were filtered through a 0.22-µm filter before detection.

### Construction of plasmids

For 3' untranslated region (3' UTR) reporter plasmid construction, we used a previously established pCMV-Tag2A-Luc plasmid 24. The 3′ UTR of *UHRF1* was amplified by PCR from genomic DNA and constructed into the pCMV-Tag2A-Luc vector immediately downstream of the luciferase gene to generate pCMV-Luc-UHRF1-3′-UTR. The fragments of the proximal promoter regions of miR-520d (-2000bp ~ -1bp) and *UHRF1* (-1000bp ~ +700bp) were amplified from human genomic DNA and constructed into a pGL3-promoter vector. The transcription start sites of miR-520d and *UHRF1* were predicted using UCSC website (http://genome.ucsc.edu/cgi-bin/hgNear). Mutations were generated by a QuikChange site-directed mutagenesis kit (Stratagene, LA Jolla, USA) and introduced into the predicted miR-520d binding site within *UHRF1* 3'UTR relevant plasmids, the predicted miR-520d binding element in the pre-miR-520d expression plasmid (Vigene Biosciences, Rockville, USA), and the predicted KLF6 binding site within the miR-520d promoter plasmid. Plasmids were verified by Sanger sequencing prior to use.

### Luciferase reporter assays

For luciferase reporter assays, the reporter plasmids were transfected (or co-transfected with siRNAs or gene expression plasmids) into cells using Lipofectamine 2000 reagent (Invitrogen, Carlsbad, USA). Luciferase activities were measured 48 hours after transfection using a Luciferase Assay System with Reporter Lysis Buffer (E4030, Promega Corporation, USA). PGE_2_ (200 ng/mL) or supernatant treatments were performed 24 hours before luciferase activity detection.

### Colony formation assay

For colony formation assays, 1,000 cells were seeded onto 20-mm dishes. After a 2-week incubation in a humidified incubator (37 °C, 5% CO_2_), the supernatant was removed and the cells were stained with crystal violet. Subsequently, after being imaged with a light microscope, the positive colonies with > 50 µm in diameter in the dishes were counted. For colony formation assays in soft agar, after being seeded onto the agar base (0.8% agarose), 1,000 cells were suspended in 1 mL of soft agar mixture (10% FBS and 0.4% agarose) and cultured for 2-3 weeks. Colonies with more than 10 cells were counted under a microscope.

### TAM migration assay

The upper chamber of a transwell culture system (8-μm pore size, Corning, USA) was pre-coated with 50 μL Matrigel solution and incubated at 37 °C for 5 hours for gelation. TAMs in suspension (2,000 cells per well) were seeded into the pre-coated upper chamber. HepG2 cells (10^4^) in 1mL of RPMI 1640 medium mixed with isotype IgG or anti-CSF1 antibody (2 µg/mL) was added into the lower chamber. After cells were incubated for 48 hours at 37 °C with 5% CO_2_, the inner bottom surface of the upper chamber was scrubbed carefully to remove Matrigel. The cells that migrated through Matrigel appearing on the outer bottom surface of the upper chamber were fixed with 4% paraformaldehyde, stained with 0.1% crystal violet, and counted.

### Statistical analysis

Student's *t*-tests were used to compare quantitative data between two groups. One-way ANOVA with Dunnett's multiple comparisons tests were used for multiple-comparison experiments. Fisher's exact tests were used to analyze categorical data. Overall patient survival was defined from the date of diagnosis to that of disease-related death. Survival rates were estimated using the Log-rank (Mantel-Cox) test. Pearson correlation coefficient was used to calculate the correlation between the expression data for each individual. All analyses were done using SPSS software (version 16.0). P < 0.05 was considered statistically significant. In the result section, one asterisk indicated P < 0.05 and N.S. indicated P > 0.05. Error bars represented S.E.M.

## Results

### TAMs upregulate HCC UHRF1 expression via PGE_2_

**Figure 1:**
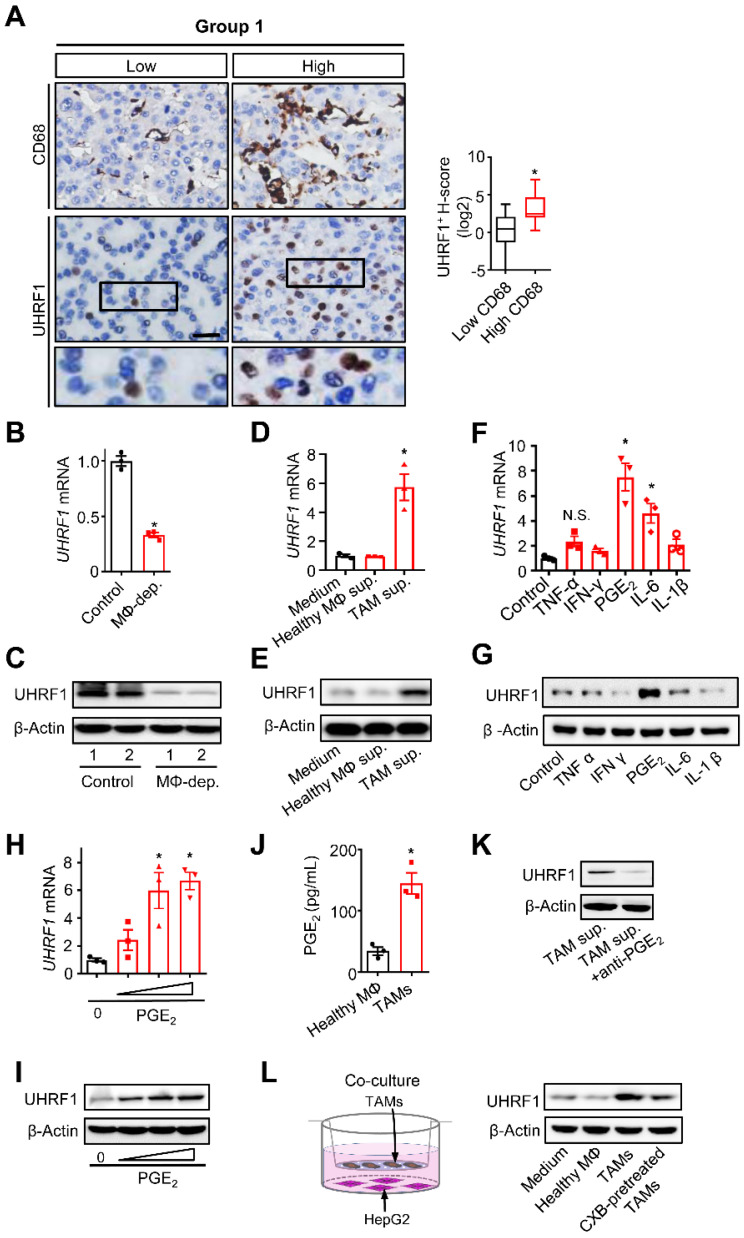
** TAMs secrete PGE_2_ to promote UHRF1 expression in HCC cells. (A)** Left: The representative images of CD68 and UHRF1 immunohistochemical staining in human HCC tissues (Group 1); bottom panel: enlargement of the black-line boxed regions. Scale bar, 50 µm. Right: Relationship between tumor UHRF1 expression and CD68^+^ macrophages in human HCC tissues. Low CD68, n = 27; High CD68, n = 26. *P = 0.003, versus Low CD68. Median, 25/75% quartiles (boxes), and Min.-Max. values (whisker) are shown. Student's *t*-test. **(B, C)** Uhrf1 mRNA** (B)** and protein levels** (C)** in murine HCC H22 xenograft tumors in the mice without (Control) or with macrophage depletion (MΦ dep.). In **C**, representative blot images (two mice per group) are shown. n = 3, *P = 0.0002. Student's *t*-test. **(D, E)** UHRF1 mRNA **(D)** and protein levels **(E)** in HepG2 cells after being incubated with the culture medium (Medium) and the supernatants of macrophages isolated from healthy volunteers (Healthy MΦ sup.) or the supernatants of human HCC TAMs (TAM sup.) for 24 hours. In **D**, n = 3, *P = 0.0065, versus Medium. One-way ANOVA with Dunnett's multiple comparisons test. **(F, G)** UHRF1 mRNA **(F)** and protein levels **(G)** in HepG2 cells after being incubated with TNF-α (10 ng/mL), IFN-γ (10 ng/mL), PGE_2_ (200 ng/mL), IL-6 (20 ng/mL), or IL-1β (10 ng/mL) for 24 hours. In **F**, n = 3, *P = 7×10^-6^, PGE_2_ versus Control; n = 3, *P = 0.00126, IL-6 versus Control. One-way ANOVA with Dunnett's multiple comparisons test. **(H**,** I)** UHRF1 mRNA **(H)** and protein levels** (I)** in HepG2 cells incubated with 0, 100, 200 or 400 ng/mL of PGE_2_ in culture medium. In **H**, n = 3, *P = 0.0025 and 0.0011 for PGE_2_ 200 and 400 ng/mL versus PGE_2_ 0 ng/mL, respectively. One-way ANOVA with Dunnett's multiple comparisons test.** (J)** PGE_2_ concentrations in the supernatants of macrophages isolated from healthy volunteers (Healthy MΦ) or in the supernatants of human HCC TAMs (TAMs). Cells were cultured with RPMI medium for 24 hours prior to measurement. n = 3, *P = 0.0039. Student's *t*-test. **(K)** UHRF1 expression in HepG2 cells cultured with human HCC TAM supernatants with anti-PGE_2_ mAb or isotype (TAM sup.). **(L)** UHRF1 expression in HepG2 cells growing in the lower chamber of a transwell co-culture system with fresh medium (Medium), macrophages isolated from healthy volunteers (Healthy MΦ), human HCC TAMs (TAMs), or celecoxib-pretreated HCC TAMs (CXB-pretreated TMAs) in the upper chamber of the transwell system.

To explore the potential relationship between TAMs and UHRF1 expression in HCC, we first quantified CD68^+^ TAMs and HCC UHRF1 expression by immunohistochemistry staining in human HCC tissues (Figure S1A and Table S1). We showed that the numbers of TAMs were significantly higher in HCC tissues than that in the paired adjacent normal tissues (Figure S1A) and the abundance of TAMs was negatively associated with patient survival (Figure S1B). In addition, we detected high levels of UHRF1 expression in HCC tissues compared to that in the paired adjacent normal tissues (Figure S1C). High levels of HCC UHRF1 expression were negatively associated with patient survival (Figure S1D). Similar results were obtained in HCC patients from The Cancer Genome Atlas (TCGA) database (https://gdc.cancer.gov/) (Figure S1E-F). The data prompted us to study a potential correlation between TAMs and UHRF1 expression in patients with HCC. Indeed, a positive correlation between TAM numbers and UHRF1 expression was found in HCC tissues (Figure 1A). The data suggest a potential biological interplay between TAMs and UHRF1. In support of this possibility, we inoculated a murine HCC cell line, H22 cells, into BALB/c mice and established xenografted tumors. We depleted macrophages with Clodronate liposomes in these H22 tumor bearing mice. This depletion resulted in a dramatic decrease in HCC Uhrf1 transcript (Figure 1B) and protein expression (Figure 1C) within the tumors. Thus, TAMs may induce UHRF1 expression in HCC cells.

To explore how macrophages induced tumor UHRF1 expression, we isolated and cultured TAMs from fresh human HCC tissues and collected TAM supernatants. These supernatants were used to culture HepG2 cells. TAM-derived supernatants, but not normal blood macrophage-derived supernatants, upregulated UHRF1 transcript (Figure 1D) and protein (Figure 1E) expression in HCC cells, indicating that TAMs may secrete factor(s) to induce UHRF1 expression. It is well known that TAMs can release multiple inflammatory molecules, including TNF-α, IFN-γ, PGE_2_, IL-6, and IL-1. We cultured HepG2 cells with these molecules and found that PGE_2_ was the most potent molecule to induce tumor cell UHRF1 transcript (Figure 1F) and protein (Figure 1G) expression. This effect was manifested in a dose-dependent manner (Figure 1H-I). As a confirmation, we detected high levels of PGE_2_ in human HCC TAMs, but not in normal macrophages (Figure 1J). Furthermore, addition of PGE_2_ neutralizing monoclonal antibody (anti-PGE_2_ mAb) into TAM supernatants abolished upregulated UHRF1 expression in tumor cells (Figure 1K). When human HCC TAMs were pretreated with celecoxib, a COX-2 specific inhibitor, which blocked PGE_2_ production, TAMs failed to stimulate tumor cell UHRF1 expression (Figure 1L). In addition to HepG2 cells, we also found that PGE_2_ and TAM supernatants stimulated UHRF1 expression in Huh7 cells, another human HCC cell line (Figure S1G-H), and anti-PGE_2_ blocked this effect (Figure S1H). Thus, TAMs induce tumor UHRF1 expression via PGE_2_.

### TAM-derived PGE_2_ upregulates UHRF1 expression by suppressing miR-520d

**Figure 2:**
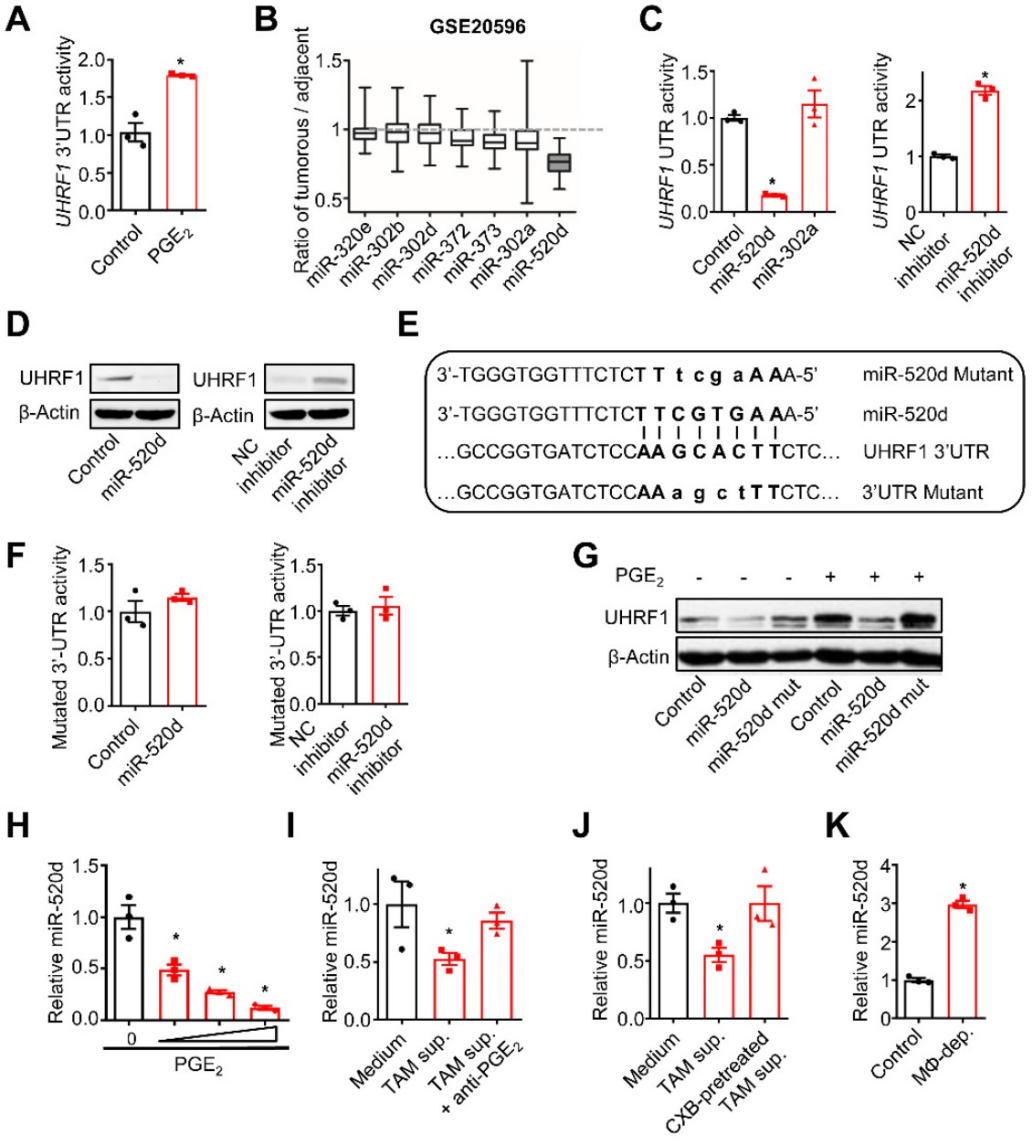
** TAM-derived PGE_2_ upregulates UHRF1 through inhibiting miR-520d. (A)** The activity of *UHRF1* 3'UTR reporter in HepG2 cells after being incubated with PGE_2_ (200 ng/mL) for 24 hours. n = 3, *P = 0.0037. Student's *t*-test. **(B)** The expression ratios of candidate microRNA levels in HCC tissues relative to their levels in the paired adjacent normal tissues. Median, 25/75% quartiles (boxes), and Min.-Max. values (whisker) are shown. 100 HCC patients from GSE20596. **(C)** Left: The activity of *UHRF1* 3'UTR reporter in HepG2 cells transfected with the expression plasmids of miR-520d, miR-302a, or nonsense control miRNA (Control). *P = 0.0025, versus Control. One-way ANOVA with Dunnett's multiple comparisons test. Right: The activity of *UHRF1* 3'UTR reporter in HepG2 cells transfected with the expression plasmids of miR-520d mimics (miR-520d inhibitor) or nonsense control miRNA inhibitor (NC inhibitor). n = 3, *P =0.0001. Student's *t*-test. **(D)** Left: UHRF1 protein levels in HepG2 cells transfected with the expression plasmids of miR-520d or nonsense control miRNA (Control). Right: UHRF1 protein levels in HEK293T cells transfected with the expression plasmids of miR-520d mimics (miR-520d inhibitor) or nonsense control miRNA inhibitor (NC inhibitor). **(E)** The predicted binding site (bold uppercase) of miR-520d in *UHRF1* 3'UTR and the designed mutations (bold lowercase) at the binding site. **(F)** Left: The activity of mutated *UHRF1* 3'UTR reporter in HepG2 cells transfected with the plasmids expressing miR-520d or nonsense control miRNA (Control). n = 3, P = 0.4935, Student's *t*-test. Right: The activity of mutated *UHRF1* 3'UTR reporter in HepG2 cells transfected with the plasmids expressing miR-520d mimics (miR-520d inhibitor) or nonsense control miRNA inhibitor (NC inhibitor). n = 3, P = 0.637, Student's *t*-test. **(G)** UHRF1 protein levels in HepG2 cells stably expressing miR-520d, miR-520d mutant (miR-520d mut), or nonsense control miRNA (Control) without or with PGE_2_ (200 ng/mL) treatment for 24 hours. **(H)** MiR-520d levels in HepG2 cells after being incubated with 0, 100, 200, 400 ng/mL of PGE_2_ for 24 hours. n = 3, *P = 5.16 × 10^-4^, 4.6 × 10^-5^, 1.1 × 10^-5^ for PGE_2_ 100, 200, 400 ng/mL versus PGE_2_ 0 ng/mL, respectively. One-way ANOVA with Dunnett's multiple comparisons test. **(I)** MiR-520d levels in HepG2 cells after being incubated with fresh medium (Medium), TAM supernatants (TAM sup.), or TAM supernatants mixed with anti-PGE_2_ mAb (2 µg/mL) (TAM sup. + anti-PGE_2_) for 24 hours. n = 3, *P = 0.001679, versus Medium. One-way ANOVA with Dunnett's multiple comparisons test.** (J)** MiR-520d levels in HepG2 cells incubated with fresh medium (Medium), TAM supernatants (TAM sup.) or the supernatants of celecoxib-pretreated TAMs (CXB-pretreated TAM sup.) for 24 hours. n = 3, *P = 0.007407, versus Medium. One-way ANOVA with Dunnett's multiple comparisons test. **(K)** MiR-520d levels in HepG2 xenograft tumors in nude mice without (Control) or with macrophage depletion (MΦ-dep.). n = 3 per group, *P = 9.52 x 10^-5^. Student's *t*-test.

To investigate the mechanisms by which TAM-derived PGE_2_ stimulates tumor UHRF1 expression, we constructed *UHRF1* 3'UTR into a luciferase reporter plasmid and cloned the *UHRF1* promoter. We observed that PGE_2_ stimulation increased the activity of *UHRF1* 3'UTR (Figure 2A), but not the activity of the *UHRF1* promoter (Figure S2A). MicroRNAs often target 3'UTR to mediate gene regulation. Using multiple microRNA prediction programs (PicTar, TargetScan, miRanda, and miRGen), we identified that miR-320e, miR-302b, miR-302d, miR-372, miR-373, miR-302a, and miR-520d could target *UHRF1* 3'UTR. Among these microRNAs, miR-520d was selected for additional studies due to its lowest expression levels in HCC (GSE20596) 25 (Figure 2B). Enforced miR-520d expression and miR-520d inhibitors (miR-520d mimics) suppressed and enhanced the 3'UTR activities (Figure 2C) and the expression of UHRF1 (Figure 2D), respectively. As a control, miR-302a had no effect on *UHRF1* 3'UTR activity (Figure 2C). These results suggest that miR-520d may target the 3'UTR of *UHRF1* and negatively regulate UHRF1 expression. To validate this, we mutated the predicted targeting sequence of *UHRF1* 3'UTR in the reporter plasmid (Figure 2E). The 3'UTR mutation abrogated the effects of miR-520d and miR-520d inhibitor on the reporter activity of *UHRF1* 3'UTR (Figure 2F), and the mutant miR-520d failed to repress spontaneous UHRF1 expression in HepG2 cells (Figure 2G). The data suggest that miR-520d binds to the predicted site in *UHRF1* 3'UTR to downregulate UHRF1 expression.

Next, we examined the regulatory relationship between TAM-derived PGE_2_ and tumor miR-520d expression. To this end, we cultured HepG2 cells with PGE_2_, TAM-derived supernatants, and TAMs. PGE_2_ treatment reduced tumor cell miR-520d expression in a dose-dependent manner (Figure 2H). Human TAM-derived supernatants (Figure 2I-J) and the co-culture with TAMs (Figure S2B) suppressed tumor cell miR-520d expression, whereas pretreatment of TAMs with anti-PGE_2_ mAb (Figure 2I) and celecoxib (Figure 2J) rescued the suppressive effect of TAMs on tumor miR-520d. Thus, TAM-derived PGE_2_ represses tumor miR-520d expression. Consistent with the *in vitro* observations, macrophage depletion by Clodronate liposomes resulted in increased tumor miR-520d levels in the HepG2 xenograft model (Figure 2K). Furthermore, overexpression of miR-520d, but not its mutant, abrogated PGE_2_-induced tumor UHRF1 upregulation (Figure 2G). Thus, TAM-derived PGE_2_ dampens miR-520d expression to promote high expression of UHRF1 in HCC cells.

### MiR-520d targets UHRF1 to control HCC progression

**Figure 3:**
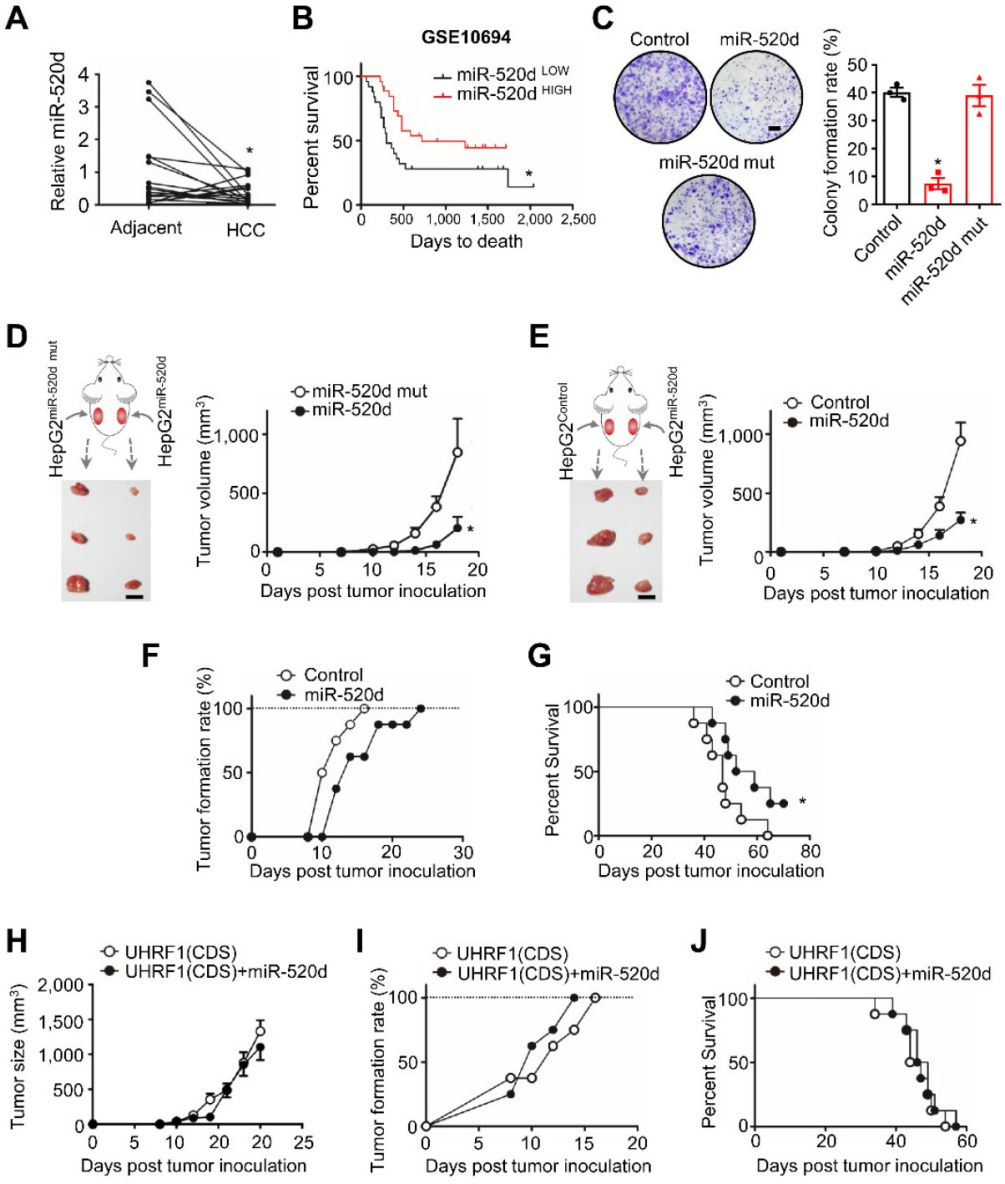
** MiR-520d targets *UHRF1* to control HCC progression. (A)** MiR-520d levels in HCC tissues and their paired adjacent normal tissues. n = 18 patients, *P = 0.0365. Student's *t-*test. **(B)** Kaplan-Meier overall survival stratified by miR-520d levels in human HCC tissues (GSE10694). n = 25 for miR-520d ^LOW^; n = 26, miR-520d ^HIGH^. *P = 0.031. Log-rank (Mantel-Cox) test. **(C)** Left: Images of colony formation assays using HepG2 cells stably expressing miR-520d, miR-520d mutant (miR-520d mut), or nonsense miRNA control (Control). Scale bar, 4 mm. Right: Quantification of colony formation rate. n = 3, *P = 1.2 x 10^-4^, versus Control. One-way ANOVA with Dunnett's multiple comparisons test. **(D)** Growth of HepG2 xenograft tumors stably expressing miR-520d or miR-520d mutant (miR-520d mut). These two types of cells were subcutaneously inoculated into the left and right posterior flanks of the same NCG mice, respectively. n = 3, *P = 0.0369. Student's *t*-test. **(E)** Effect of miR-520d on tumor growth. HepG2 cells stably expressing miR-520d or nonsense miRNA control (Control) were inoculated into the left and right posterior flank of the same NCG mice, respectively. n = 3, *P = 0.0162. Student's *t*-test.** (F, G)** Tumor formation rate **(F)** and survival of nude mice **(G)**. Mice were subcutaneously inoculated with 10^7^ HepG2 cells stably expressing miR-520d or nonsense miRNA control (Control). n = 8 per group, *P = 0.038, Log-rank Mantel-Cox test. **(H-J)** Role of UHRF1 in tumor growth** (H)**, tumor formation rate **(I)** and mouse survival **(J)**. Nude mice were inoculated with 10^7^ HepG2 cells stably expressing UHRF1 coding sequence along with nonsense miRNA control (UHRF1 CDS) or miR-520d (UHRF1 CDS + miR-520d). n = 8 per group, P > 0.05.

We next examined functional relevance of the interaction between miR-520d and UHRF1 in HCC. To this end, we quantified and compared the levels of miR-520d in human HCC tissues and the paired adjacent tissues (18 patients) (Figure 3A). Real-time PCR showed that the levels of miR-520d were lower in HCC tissues than the paired adjacent tissues (Figure 3A). Low levels of miR-520d expression were associated with short disease-free survival (GSE10694) 26 (Figure 3B). Moreover, miR-520d overexpression in HepG2 cells suppressed anchorage-independent colony formation (Figure 3C). To examine the effect of miR-520d on tumor growth *in vivo*, we subcutaneously inoculated HepG2 cells overexpressing miR-520d, miR-520d mutants, and control vectors into the two posterior flanks of the same NOD-Prkdc^em26Cd52^II2rg^em26Cd22^/Nju (NCG) mice. The tumors expressing miR-520d were smaller than the tumors expressing mutant miR-520d (Figure 3D) or control vectors (Figure 3E). We obtained the similar inhibitory effects of forced miR-520d expression on tumor growth when miR-520d expressing tumor cells and control vector expressing tumor cells were inoculated into different nude mice (Figure S3A). Forced miR-520d expression also reduced the incidence of tumor formation (Figure 3F) and improved mouse survival (Figure 3G). To determine whether miR-520d suppressed HCC progression through targeting *UHRF1*, we first tested and validated the oncogenic role of UHRF1 in the HepG2 model. We established HepG2 cells stably expressing specific short hairpin RNA against *UHRF1* (shUHRF1) and inoculated these cells into nude mice. As expected, shUHRF1 resulted in reduced tumor volume (Figure S3B) and increased mouse survival (Figure S3C). Given that miR-520d targeted *UHRF1* 3'UTR, we cloned the *UHRF1* coding sequence (CDS) without 3'UTR, established HepG2 cells stably expressing both UHRF1 CDS and miR-520d, and inoculated these cells into nude mice. We observed that miR-520d overexpression had no effects on tumor growth (Figure 3H), tumor incidence (Figure 3I), and mouse survival (Figure 3J). Thus, miR-520d inhibits HCC progression via targeting *UHRF1* 3'UTR.

### UHRF1 suppresses KLF6 through H3K9 methylation to promote HCC development

**Figure 4:**
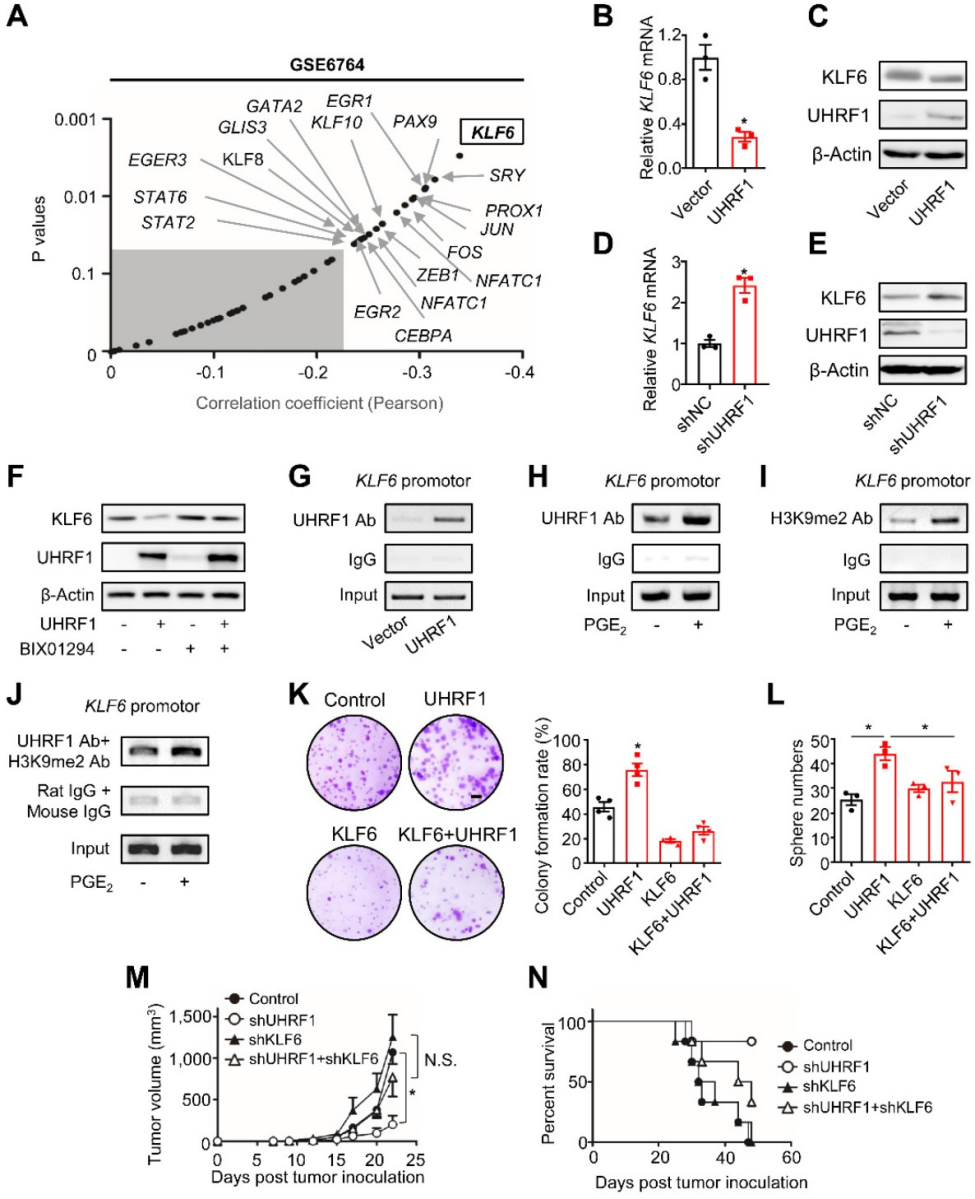
**UHRF1 inhibits KLF6 through H3K9 methylation to promote HCC progression. (A)** Pearson correlation analysis on *UHRF1* with 49 putative transcription factors using the data from GSE6764. Unshaded area covers the genes (black dots indicated by arrows) with statistical significance (*r* > -0.2, P < 0.05). *P_(*KLF6*)_ = 0.002906. Pearson product-moment correlation coefficient.** (B, C)** KLF6 mRNA **(B)** and protein levels **(C)** in L02 cells (human immortalized normal liver cell line) overexpressing UHRF1 or control vector (Vector). In **B**, n = 3, *P = 0.0043. Student's *t*-test.** (D, E)** KLF6 mRNA **(D)** and protein levels** (E)** in HepG2 cells overexpressing shUHRF1 or nonsense control shRNA (shNC). In **D,** n = 3, *P = 0.0023. Student's *t*-test.** (F)** KLF6 protein levels in HepG2 or UHRF1-overexpressing HepG2 cells treated without or with BIX01294 for 24 hours.** (G)** UHRF1 occupancy on the *KLF6* promoter in HepG2 cells transfected with UHRF1 or control vector (Vector).** (H, I)** UHRF1** (H)** and H3K9me2 **(I)** abundance on the *KLF6* promoter in HepG2 cells incubated without or with PGE_2_ (200 ng/mL) for 24 hours.** (J)** Re-ChIP assays with the antibodies against UHRF1 and H3K9me2 to analyze their co-localization on the *KLF6* promoter in HepG2 cells that were incubated without or with PGE_2_ (200 ng/mL) for 24 hours prior to use.** (K)** Left: Images of colony formation assays using 10^4^ HepG2 cells stably expressing empty vector (Control), UHRF1, KLF6, or both (KLF6 + UHRF1). Scale bar, 4 mm. Right: Quantification of colony formation rates. n = 3, *P = 0.00706, versus Control. One-way ANOVA with Dunnett's multiple comparisons test.** (L)** Sphere formation of 2,000 HepG2 cells stably expressing empty vector (Control), UHRF1, KLF6, or both (KLF6 + UHRF1) after being cultured for 2 weeks. n = 3, *P = 0.002, UHRF1 versus Control; *P = 0.049, UHRF1 versus KLF6 + UHRF1. One-way ANOVA with Dunnett's multiple comparisons tests.** (M, N)** Tumor growth **(M)** and overall survival **(N)** of nude mice inoculated with 10^7^ HepG2 cells stably expressing control vector (Control), shUHRF1, shKLF6, or both (shUHRF1+ shKLF6). n = 8 per group. In **M**, *P = 0.006536, shUHRF1 versus Control. One-way ANOVA with Dunnett's multiple comparisons test.

UHRF1 may inhibit tumor suppressor genes to promote tumor progression 27. To identify the potential targets of UHRF1 in HCC cells, we analyzed gene expression profiles in the Kyoto Encyclopedia of Genes and Genomes (KEGG) Pathways in Cancer (GSE6764) 28. Among a set of genes that were negatively correlated with *UHRF1* expression, *KLF6* had the highest coefficient (Figure 4A). Such a reverse correlation between *UHRF1* and *KLF6* levels was also observed in the tumors from HCC patients in TCGA (Figure S4A). KLF6, a zinc finger transcription factor, is a tumor suppressor that controls cell proliferation, differentiation, and migration 29. We wondered whether UHRF1 affected KLF6 expression in HCC. Overexpression of UHRF1 reduced KLF6 transcript (Figure 4B) and protein levels in HepG2 (Figure 4C) and Huh7 cells (Figure S4B), while knocking-down UHRF1 with shUHRF1 increased KLF6 expression (Figure 4D-E and Figure S4C). The data indicates that UHRF1 negatively regulates KLF6 expression. In contrast, UHRF1 had no effects on expression of other reverse correlated genes, including *PTEN*, *RUNX3*, *RASSF1* (Figure S4D). In addition, we connected UHRF1 regulation on KLF6 to functional activities of miR-520d, PGE_2_, and TAMs in HCC. We found that overexpression of miR-520d (Figure S4E) and treatment with PGE_2_ (Figure S4F), respectively, enhanced and reduced KLF6 transcript and protein expression in HepG2 (Figure S4E-F) and Huh7 (Figure S4F) cells. Moreover, overexpression of miR-520d abolished the inhibiting effect of PGE_2_ on KLF6 expression (Figure S4G). Furthermore, the supernatants from TAMs, but not from normal macrophages, reduced KLF6 expression in HepG2 cells (Figure S4H). This effect was partially, but significantly, reversed by COX-2 inhibitor (Figure S4H). Together, these results indicate that UHRF1 suppresses KLF6 expression and may form a regulatory loop with PGE_2_ and miR-520d in HCC cells.

UHRF1 can recruit histone H3 lysine 9 (H3K9) methyltransferase to propagate methylation of H3K9 in nucleosomes 12, 13, 30-33*.* In line with this, we observed that BIX01294, an inhibitor of H3K9 methyltransferase G9a, abolished the repressive effect of UHRF1 on KLF6 expression (Figure 4F). Similarly, knockdown of G9a also abrogated UHRF1's repressive effects on KLF6 expression in both HepG2 and Huh7 cells (Figure S4I-J). In addition, the chromatin immunoprecipitation (ChIP) assay demonstrated that UHRF1 bound to the *KLF6* promoter (Figure 4G) and its binding abundance on *KLF6* was increased by PGE_2_ (Figure 4H). Meanwhile, PGE_2_ also enhanced the presence of methylated H3K9 in the *KLF6* promoter (Figure 4I). Consistent with the fact that UHRF1 silencing (knockdown) by CRISPR/Cas9 (Figure S4K; see Materials and Methods for details) abolished the inhibitory effect of PGE_2_ on KLF6 (Figure S4L), such UHRF1 silencing also reduced the abundance of H3K9me2 in *KLF6* promoter region (Figure S4M), indicating that UHRF1 promotes H3K9me2 in *KLF6* promoter region and therefore represses KLF6 expression in response to PGE_2_ treatment. Furthermore, re-CHIP assay revealed that methylated H3K9 was co-localized with UHRF1 within the *KLF6* promoter (Figure 4J). As PGE_2_ and UHRF1 had no effects on *KLF6*'s DNA methylation levels (Figure S4N), UHRF1 mediates H3K9 methylation of the *KLF6* promoter to repress KLF6 expression.

Finally, we tested whether the regulatory role of UHRF1 in KLF6 is functionally critical for HCC progression. To this end, UHRF1, KLF6, or both UHRF1 and KLF6 were ectopically expressed in HepG2 cells. Again, overexpression of UHRF1 and KLF6, respectively, increased and decreased tumor colony formation and sphere formation (Figure 4K-L). The pro-tumor effect of ectopic UHRF1 was abolished by KLF6 overexpression (Figure 4K-L). To support the biological interaction between UHRF1 and KLF6 *in vivo*, we used specific shRNAs to knock down UHRF1, KLF6, or both UHRF1 and KLF6 in HepG2 cells. We inoculated these cells into nude mice. In line with our previous data (Figure S3B-C), silencing UHRF1 reduced tumor growth and prolonged mouse survival. As expected, silencing KLF6 enhanced tumor growth and shortened mouse survival. Interestingly, shKLF6 abolished the effects of shUHRF1 on tumor growth and mouse survival (Figure 4M-N). In patients with HCC, increased UHRF1 expression (Figure S1C) and decreased KLF6 expression (Figure S4O) were detected in HCC tissues compared to paired adjacent tissues. Together, these results support that UHRF1 suppresses KLF6 to promote HCC development.

### KLF6 and miR-520d form a molecular network in HCC

**Figure 5:**
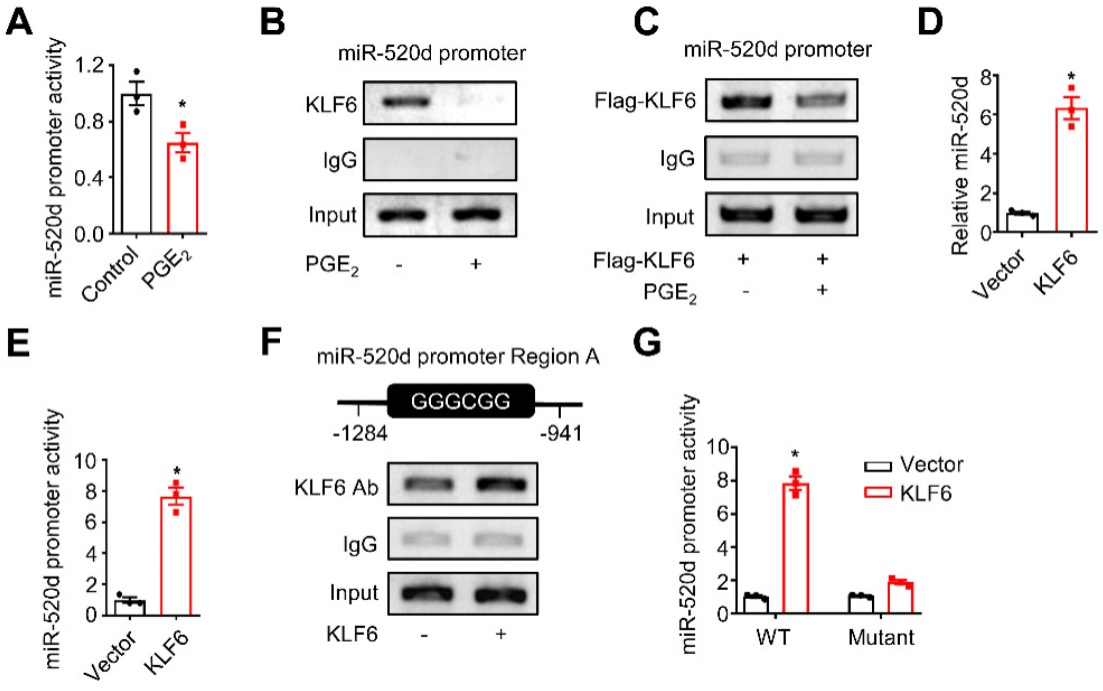
** KLF6 and miR-520d form a molecular network in HCC. (A)** The activity of the miR-520d promoter in HepG2 cells after being incubated without or with PGE_2_ (200 ng/mL) for 24 hours. n = 3, *P = 0.0326. Student's *t*-test.** (B)** ChIP assays showing occupancy of endogenous KLF6 on the miR-520d promoter in HepG2 cells after being incubated without or with PGE_2_ (200 ng/mL) for 24 hours.** (C)** ChIP assays showing occupancy of exogenous KLF6 on the miR-520d promoter in HepG2 cells after being incubated without or with PGE_2_ (200 ng/mL) for 24 hours. Flag-KLF6, KLF6 protein fused with a Flag tag at N-terminus.** (D)** MiR-520d levels in HepG2 cells stably expressing empty vector (Vector) or KLF6. n = 3, *P = 0.0008. Student's *t*-test.** (E)** The activity of the miR-520d promoter in HepG2 cells stably expressing empty vector (Vector) or KLF6. n = 3, *P = 0.000339. Student's *t*-test.** (F)** ChIP assays showing KLF6 occupancy on the “GC box” (Region A) of the miR-520d promoter in HepG2 cells stably expressing empty vector or KLF6.** (G)** The reporter activities of the miR-520d promoter (WT) or the mutated miR-520d promoter (Mutant) where the GGGCGG (Region A) sequence was altered (see Supplementary Fig. S5B) in HepG2 cells stably expressing empty vector (Vector) or KLF6. n = 3, *P = 8.05 x 10^-5^. Student's *t*-test.

We demonstrated that TAM-derived PGE_2_ stimulated tumor UHRF1 expression (Figure 1) via blocking miR-520d expression (Figure 2), loss of miR-520d expression led HCC progression via tumor UHRF1 (Figure 3), and tumor UHRF1 targeted tumor repressor KLF6 (Figure 4). Accordingly, we wondered whether KLF6 was involved in the regulation of the suppressive effect of PGE_2_ on tumor miR-520d. PGE_2_ treatment inhibited the miR-520d promoter reporter activities in both HepG2 (Figure 5A) and Huh7 cells (Figure S5A). In line with this, ChIP assays showed that PGE_2_ reduced the occupancy of KLF6 in the miR-520d promoter in HepG2 cells without (Figure 5B) or with (Figure 5C) ectopic KLF6 expression. Furthermore, the suppressive effect of PGE_2_ on miR-520d expression was abolished by UHRF1 depletion (Figure S5B). The data suggest an interaction between KLF6 and tumor miR-520d in the context of PGE_2_ biological activity in HCC. To dissect the mechanistic relationship between KLF6 and miR-520d, we tested the possibility that KLF6 promoted miR-520d transcription. Overexpression of KLF6 upregulated miR-520d transcripts (Figure 5D). The upstream region in the miR-520d promoter contains a “GC box” and a “CACCC” element, which KLF6 preferentially recognizes 29, 34 (Figure S5C). We fused the miR-520d promoter with a luciferase coding sequence (Figure S5C) and constructed a specific miR-520d promoter reporter. We showed that KLF6 overexpression increased the miR-520d reporter activity (Figure 5E). ChIP assays showed that KLF6 bound to the “GC box” (Figure 5F), but not the “CACCC” element within the miR-520d promoter (Figure S5D). Mutating the “GC box” (Figure S5C) abolished the stimulatory role of KLF6 in the miR-520d promoter (Figure 5G). Thus, KLF6 binds to the miR-520d promoter and stimulates miR-520d expression, and in turn, suppresses UHRF1 expression. Therefore, our data demonstrates that KLF6 and miR-520d form a regulatory molecular network in HCC.

### TAMs promote HCC progression via the UHRF1 and CSF1 network

**Figure 6:**
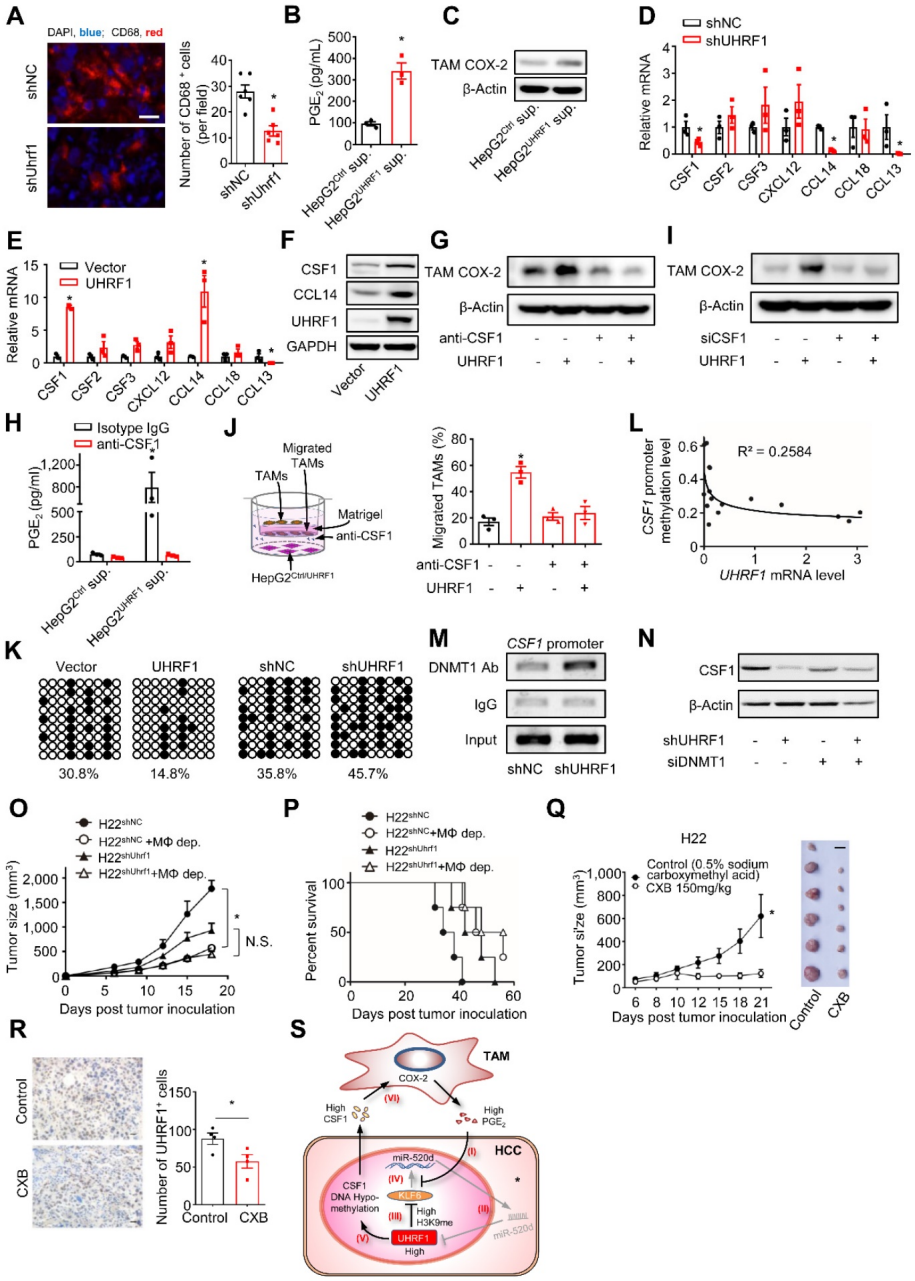
** TAMs promote HCC progression via the UHRF1 and CSF1 network. (A)** Left: Immunofluorescent staining of CD68^+^ TAMs (red) in xenograft H22 tumors expressing nonsense control shRNA (shNC) or shUhrf1. The nuclei (blue) were stained by DAPI. Scale bar, 50 µm. Right: CD68 positive cells per field. Six randomly selected microscopic fields per sample. n = 6 samples per group, *P = 0.0007. Student's *t*-test. **(B, C)** PGE_2_ secretion** (B)** and COX-2 expression** (C)** in human HCC TAMs. TAMs were incubated with the supernatants of HepG2 cells expressing control vector (HepG2^Ctrl^ sup.) or UHRF1 (HepG2^UHRF1^ sup.) for 24 hours. n = 3, *P = 0.0031. Student's *t*-test. **(D, E)** The relevant mRNA levels in HepG2 cells stably expressing nonsense control shRNA (shNC) and shUHRF1 **(D)**, or expressing empty vector (Vector) and UHRF1 **(E)**. n = 3, *P < 0.05, versus corresponding control (shNC or Vector). Student's *t*-test. **(F)** CSF1 and CCL14 protein levels in HepG2 cells stably expressing empty vector (Vector) or UHRF1. **(G)** COX-2 protein levels in TAMs incubated with the supernatants from HepG2 cells or UHRF1-overexpressing HepG2 cells. HepG2 cells were treated without or with CSF1 neutralizing antibody (anti-CSF1, 2 μg/mL) for 24 hours. **(H)** PGE_2_ secretion from human HCC TAMs. TAMs were incubated with the supernatants from HepG2 cells or UHRF1-overexpressing HepG2 cells that were treated without or with anti-CSF1 (2 µg/mL) for 48 hours. Isotype IgG as an antibody control. n = 3, *P = 0.03117. Student's *t*-test. **(I)** COX-2 protein levels in human HCC TAMs incubated with the supernatants from HepG2 cells or UHRF1-overexpressing HepG2 cells that were treated without or with siRNA against CSF1 (siCSF1) for 48 hours. **(J)** Left: Schematics showing transwell assays analyzing human HCC TAMs. Human TAMs were seeded in the upper chamber. HepG2 cells (HepG2^Ctrl^) or UHRF1-overexpressing HepG2 cells (HepG2^UHRF1^) were seeded in the lower chamber. TAMs were cultured in the medium without or with anti-CSF1 (2 μg/mL). Isotype IgG as an antibody control. Right: Percentages of migrated TAMs relative to total TAMs. n = 3 with replicates. *P = 1.39 × 10^-4^, versus Control (HepG2^Ctrl^ in medium without anti-CSF1). One-way ANOVA with Dunnett's multiple comparisons test. **(K)** DNA methylation of CpG islands in the *CSF1* promoter in HepG2 cells stably expressing control vector (Vector), UHRF1, shNC or shUHRF1. Closed circles indicate methylated CpGs. Open circles represent unmethylated CpGs. Percentage of DNA methylation (methylated CpGs/ total CpGs) is given at the bottom of each panel. **(L)** Pearson correlation between *UHRF1* mRNA levels and *CSF1* promoter methylation levels in human HCC tissues. 16 HCC patients. **(M)** ChIP assay showing DNMT1 abundance on the *CSF1* promoter in HepG2 cells stably expressing shNC or shUHRF1. **(N)** CSF1 protein levels in HepG2 cells and shUHRF1-expressing HepG2 cells that were transfected without or with siRNA against *DNMT1* (siDNMT1). **(O, P)** Tumor growth** (O)** and overall survival **(P)** of the mice. Mice were subcutaneously inoculated with H22 cells stably expressing shUhrf1 (H22^shUhrf1^) or shNC (H22^shNC^). Half of the mice in each group were intraperitoneally injected with Clodronate liposomes to deplete macrophages (H22^shUhrf1^ + MΦ-dep., and H22^shNC^ + MΦ-dep.). n = 4 per group. In *O*, *P = 0.0006, H22^shNC^ + MΦ-dep. versus H22^shNC^; P = 0.0740 (N.S.), H22^shUhrf1^ + MΦ-dep. versus H22^shUhrf1^; Student's *t*-test. In *P*, *P = 0.0169, H22^shNC^ + MΦ-dep. versus H22^shNC^; P = 0.1753, H22^shUhrf1^ + MΦ-dep. versus H22^shUhrf1^; Log-rank (Mantel-Cox) test. **(Q)** Effect of celecoxib on tumor growth. H22 cells were inoculated into the left posterior flank of the Balb/c mice. The tumor bearing mice were then treated with daily oral administration of celecoxib (CXB, 150 mg/kg, n = 6) or solvent (Control, n = 6). Left panel: once tumors started growing, their sizes were measured twice weekly and tumor volume was calculated. Right panel: photographs of isolated tumors from each group. *P = 0.042. Student's *t*-test. **(R)** UHRF1 staining analysis of tissue sections from celecoxib(CXB)-treated and solvent(Control)-treated H22 tumor bearing mice (n = 6 per group). Scale bar, 1 cm. *P = 0.022. Student's *t*-test. **(S)** Schematic model showing the interactions between TAMs and HCC cells. TAMs produce and release PGE_2_ into the tumor microenvironment. (I) PGE_2_ inhibits miR-520 transcription by dissociating KLF6 from the miR-520 promoter. (II) Reduced miR-520 permits UHRF1 upregulation. (III) High-level UHRF1 epigenetically suppresses KLF6 expression via H3K9 hypermethylation. (IV) Dampened KLF6 lowers miR-520, thus allowing further elevation of UHRF1 protein level. (V) Concurrently, high-level UHRF1 epigenetically promotes CSF1 expression via DNA hypomethylation. (VI) CSF1 secreted from HCC cells promotes COX-2 expression in TAMs, leading to macrophage tumor infiltration and activation. The upregulated COX-2 in TAMs stimulates additional PGE_2_ production**.**

TAMs stimulated tumor UHRF1 expression and were positively associated with tumor UHRF1 expression in HCC (Figure 1). We wondered whether and how tumor UHRF1 impacted TAMs in HCC. To address this, we quantified TAMs in mice bearing shUhrf1-expressing H22 tumors. We detected reduced TAMs in shUhrf1-expressing tumors compared to the control tumors (Figure 6A). The data suggest that tumor UHRF1 may affect TAMs. To explore how tumor UHRF1 regulated TAMs, we cultured human HCC TAMs with the supernatants from UHRF1-overexpressing HepG2 cells. We found that UHRF1-overexpressing HepG2 cells promoted but CRISPR/Cas9-mediated UHRF1-knockdown cells inhibited PGE2 production (Figure 6B and Figure S6A) and COX-2 expression (Figure 6C and Figure S6B) in human TAMs. The results indicate that UHRF1-overexpressing and UHRF1-knockdown HCC cells could alter TAMs via soluble factor(s). We screened a known list of genes, which promote and activate macrophage differentiation and trafficking, in HepG2 cells expressing shUHRF1 (Figure 6D) and enforced UHRF1 (Figure 6E). We found that expression of *CSF1* and *CCL14* was negatively and positively altered in HepG2 cells expressing shUHRF1 (Figure 6D) and ectopic UHRF1 (Figure 6E), respectively. Western blot confirmed high levels of CSF1 and CCL14 protein in UHRF1 expressing tumor cells (Figure 6F). We blocked CSF1 (Figure 6G) and CCL14 (Figure S6C) with specific neutralizing antibodies (anti-CSF1 and anti-CCL14) in HepG2 cells stably expressing UHRF1, collected these cell supernatants, and cultured TAMs with these cell supernatants. We observed that anti-CSF1 (Figure 6G), but not anti-CCL14 (Figure S6C), abolished the stimulatory effect of UHRF1 on TAM COX-2 expression (Figure 6G) and PGE2 production (Figure 6H) in TAMs. Similar results were obtained when CSF1 (Figure 6I) and CCL14 (Figure S6D) were genetically knocked down by specific siRNAs. In addition, macrophages efficiently migrated toward the culture supernatants from enforced UHRF1-expressing tumor cells compared to the controls (Figure 6J). This effect was abolished by anti-CSF1 treatment (Figure 6J). Altogether, the data indicates that UHRF1 promotes tumor cell CSF1 production, which in turn assists macrophage tumor trafficking and activation.

High expression of CSF1 correlates with poor prognosis in a variety of cancer types 6, 35-38. We confirmed this correlation in HCC patients from the TCGA database (https://gdc.cancer.gov/) (Figure S6E). To investigate the mechanism by which UHRF1 induced CSF1, given the epigenetic role of UHRF1, we assessed DNA methylation and histone methylation within the *CSF1* promoter. UHRF1 overexpression and knocking-down led to hypomethylation and hypermethylation of the CpG islands in the *CSF1* promoter, respectively (Figure 6K). Such a reverse correlation between UHRF1 levels and the methylation of the *CSF1* promoter was also observed in the tumors from HCC patients (Figure 6L). The results suggest that UHRF1 negatively regulates DNA methylation of the *CSF1* promoter to control CSF1 expression. Supporting this, CSF1 downregulation resulting from UHRF1 knocking-down was reversed by 5-Aza-2'-deoxycytidine (5-Aza) (Figure S6F), an inhibitor of DNA methyltransferase, but not by BIX01294, an inhibitor of histone methyltransferase (Figure S6G). Moreover, UHRF1 did not affect methylation of H3K9 on the CSF1 promoter (Figure S6H), indicating that DNA methylation, but not histone methylation, mediates UHRF1-controlled CSF1 expression. In further support, ChIP assay demonstrated that shUHRF1 increased DNMT1 association with the CSF1 promoter (Figure 6M), while siDNMT1 reversed CSF1 downregulation caused by shUHRF1 (Figure 6N and Figures S6I). In addition to that UHRF1 silencing (by CRISPR/Cas9) or miR-520d overexpression both abolished the promoting effect of PGE_2_ on CSF1 (Figure S6J-K), UHRF1 silencing also strengthened the presence of DNMT1 in *CSF1* promoter region (Figure S6L), suggesting that UHRF1 promotes the dissociation of DNMT1 from *CSF1* promoter region and therefore enhances CSF1 expression in response to PGE_2_. Therefore, UHRF1 induces *CSF1* through demethylating its promoter via reducing DNMT1 association with the *CSF1* promoter. Given that UHRF1 knockdown and overexpression respectively enhanced and reduced DNMT1's protein levels but not mRNA levels (Figure S6M), UHRF1 induces *CSF1* likely through demethylating its promoter via decreasing the amount of DNMT1 protein, thus reducing DNMT1 association with *CSF1* promoter. Taken together, our data supports the notion that in HCC, UHRF1 promotes CSF1 expression and CSF1 in turn recruits and activates TAMs, leading to enhanced PGE_2_ production. The data suggest a mutually enforced interaction between macrophages and tumor UHRF1 in HCC.

To further demonstrate the functional relevance of the interaction between macrophages and tumor UHRF1 *in vivo*, we inoculated shUHRF1-expressing H22 cells into BALB/c mice with or without macrophage depletion. As expected, knockdown of tumor UHRF1 or macrophage depletion comparably slowed down tumor growth (Figure 6O) and enhanced mouse survival (Figure 6P). Simultaneous knockdown of tumor UHRF1 and macrophage depletion did not have additional effect on tumor progression and mouse survival (Figure 6O-P). Liver tumor microenvironment is characterized by high-level expression of immunosuppressive factors including COX-2 and its product 39, PGE_2_, which induced UHRF1 expression and thereby promotes cancer growth. We hypothesized that COX-2 inhibitor celecoxib might represent a useful approach for treatment of liver cancer. To determine the effect of celecoxib on tumor growth, H22 cells were subcutaneously injected into BALB/c mice. Celecoxib-treated mice had significantly smaller tumors compared to the control mice (Figure 6Q). Consistently, fewer number of UHRF1 positive cancer cells were observed in the celecoxib-treated tumors (Figure 6R). Thus, altogether, our data has defined a relatively specific cellular, molecular, and functional crosstalk between TAMs and HCC and its relevance in promoting HCC progression (Figure 6S).

## Discussion

Crosstalk between inflammation and tumor epigenetics is thought to contribute to HCC progression. TAMs are a main type of tumor infiltrating inflammatory cells. However, the potential interplay between TAMs and the tumor epigenetic regulatory network remains elusive in patients with HCC. Here, we have investigated the molecular, cellular, functional, and clinical relationship between TAMs and aberrantly high UHRF1 expression in HCC. Among a plethora of inflammatory molecules derived from TAMs, PGE_2_ affects HCC growth 40, 41, facilitates tumor metastasis 42, regulates immune evasion 43, and promotes drug resistance 44, 45. The breadth of biological activities of PGE_2_ raises the possibility that PGE_2_ may be involved in the regulation of tumor epigenetics. In support of this, we have observed that TAM-derived PGE_2_ controls tumor UHRF1, an important epigenetic coordinator of DNA methylation and histone modifications, and affects HCC progression. Previous studies have reported the association of inflammatory cytokines IL-1 and IL-6 with tumor epigenetic modifications 7, 8, 46. Our work has identified a novel role of TAM-derived PGE_2_ in HCC pathology and supports the notion that HCC TAMs shape HCC progression by targeting tumor epigenome through PGE_2_. We have shown that TAMs alter tumor UHRF1 expression via PGE_2_ in HCC. To elucidate the mechanism by which TAM-derived PGE_2_ controls UHRF1 expression, we have demonstrated that PGE_2_ promoted the dissociation of KLF6, a transcription factor and a tumor suppressor, from the miR-520d promoter, thereby decreasing miR-520d level. This, in turn, elevated UHRF1 expression. Interestingly, increased UHRF1 epigenetically suppresses tumor KLF6 expression. Thus, we have defined a reciprocal interactive loop among PGE_2_, KLF6, miR-520d, and UHRF1 in HCC. It appears that this loop serves to ensure a robust and sustained oncogenic UHRF1 expression in HCC cells.

Emerging evidence reveals that tumor epigenetic alterations impact immune cell components in the tumor microenvironment. For instance, EZH2, a histone 3 lysine 27 trimethylation regulator, mediates epigenetic silencing of tumor CXCL9 and CXCL10, two Th1-type chemokines, and consequently represses effector T cell trafficking into the tumor microenvironment 47. Along this line, our data demonstrates that high HCC UHRF1 causes increased tumor CSF1 production by reducing DNMT1-mediated DNA methylation in the *CSF1* promoter; subsequently, tumor CSF1 promotes TAM tumor infiltration and boosts TAM COX-2 expression and PGE_2_ production. Thus, TAM PGE_2_ and tumor UHRF1 form a positive circuit, which contributes to cultivating a potent inflammatory microenvironment. Our findings highlight a previously unappreciated oncogenic crosstalk between TAMs and tumor epigenome in patients with HCC.

Given the functional importance of UHRF1 during oncogenesis in multiple types of cancer, much effort has been devoted to identifying genetic targets of UHFR1. Our work has identified *KLF6* and *CSF1*, two new target genes of UHRF1 in HCC. Interestingly, KLF6 and CSF1 are differentially regulated by UHRF1 through distinct epigenetic modulations: histone methylation for KLF6 and DNA methylation for CSF1. Furthermore, we have shown that KLF6 serves as a regulatory component of the interactive loop between TAMs and HCC to control HCC UHRF1 expression. Although KLF6 and CSF1 are differentially regulated by UHRF1, their regulatory mechanisms are functionally orchestrated to help UHRF1 fulfill its tumor-promoting roles in the course of HCC progression. Thus, we have generated new mechanistic insights into UHRF1 oncogenic functions. Applicably, our work suggests multiple potential therapeutic targets in patients with HCC. In light of its oncogenic functions in different types of cancer 48, 49, including HCC, small molecule inhibitors targeting UHRF1 may have therapeutic potential 50, 51. In addition, as miR-520d represses UHRF1, miR-520d may be a therapeutic surrogate of UHRF1.

COX-2 expression in tumor cells is correlated with tumorigenesis in HCC 52, 53. Higher COX-2 expression is associated with shorter disease-free survival in HCC patients 54. The chemopreventive effect of celecoxib was reported in animal models of diethylnitrosamine-induced HCC, probably through the sustained inhibition of Akt and JNK-c-Jun survival pathways 40, 55, 56. However, much less is known about the key role of COX-2 expression in TAMs. Our data revealed that TAMs COX-2 acts as a critical link between inflammatory-related cytokine(s) and cancer cells' epigenetics. Celecoxib, a COX-2 inhibitor, reduced PGE_2_-induced UHRF1 expression in a miR-520d-dependent manner in HCC cells, suggesting that celecoxib might be potentially useful for HCC treatment.

In summary, we have shown that the crosstalk between TAMs and HCC cells impacts HCC progression through a molecular loop. TAM-derived PGE_2_ stimulates HCC UHRF1 expression by suppressing miR-520d. This suppression of miR-520d is achieved through (1) UHRF1 reducing KLF6 via H3K9 methylation and (2) PGE_2_ constraining KLF6's access to the miR-520d promoter. Meanwhile, UHRF1 induces DNA hypomethylation of the *CSF1* promoter, promoting CSF1 expression, thereby leading to TAM recruitment and activation. The latter results in robust and sustained PGE_2_ production in the tumor microenvironment. Thus, a reciprocal promoting loop between TAMs and HCC cells is formed to foster a self-enhancing oncogenic microenvironment in HCC.

## Supplementary Material

Supplementary figures and tables.

## Abbreviations

anti-PGE_2_ mAb: PGE_2_ neutralizing monoclonal antibody
CDS: coding sequence
CHIP: chromatin immunoprecipitation
CRISPR/Cas9: clustered regularly interspaced short palindromic repeats associated nuclease Cas9
CXB: celecoxib
CXB-pretreated TAM sup: supernatants of celecoxib-pretreated TAMs
DNMT1: DNA methyltransferase 1
ELISA: enzyme-linked immunosorbent assay
H3K9: histone H3 lysine 9
HCC: hepatocellular carcinoma
IHC: immunohistochemistry
KEGG: kyoto encyclopedia of genes and genomes
miR-520d mut: miR-520d mutant
MΦ-dep: macrophage depletion
NC inhibitor: control miRNA inhibitor
shNC: shRNA nonsense control
shKLF6: short hairpin RNA against *KLF6*
shUHRF1: short hairpin RNA against *UHRF1*
TAMs: tumor-associated macrophages
TAM sup: TAM supernatants
TCGA: the cancer genome atlas
TMA: tissue microarrays
UHRF1: ubiquitin-like with PHD and ring finger domains 1
3'UTR: 3' untranslated region

## References

[1] Torre LA, Bray F, Siegel RL, Ferlay J, Lortet-Tieulent J, Jemal A (2015). Global cancer statistics, 2012. CA: a cancer journal for clinicians.

[2] Galun E (2016). Liver inflammation and cancer: The role of tissue microenvironment in generating the tumor-promoting niche (TPN) in the development of hepatocellular carcinoma. Hepatology.

[3] Kornblihtt AR (2017). Epigenetics at the base of alternative splicing changes that promote colorectal cancer. The Journal of clinical investigation.

[4] Qian BZ, Pollard JW (2010). Macrophage diversity enhances tumor progression and metastasis. Cell.

[5] Zou W (2005). Immunosuppressive networks in the tumour environment and their therapeutic relevance. Nature reviews Cancer.

[6] DeNardo DG, Brennan DJ, Rexhepaj E, Ruffell B, Shiao SL, Madden SF (2011). Leukocyte complexity predicts breast cancer survival and functionally regulates response to chemotherapy. Cancer discovery.

[7] Gasche JA, Hoffmann J, Boland CR, Goel A (2011). Interleukin-6 promotes tumorigenesis by altering DNA methylation in oral cancer cells. Int J Cancer.

[8] Rokavec M, Oner MG, Hermeking H (2016). lnflammation-induced epigenetic switches in cancer. Cell Mol Life Sci.

[9] Bronner C, Achour M, Arima Y, Chataigneau T, Saya H, Schini-Kerth VB (2007). The UHRF family: oncogenes that are drugable targets for cancer therapy in the near future?. Pharmacol Ther.

[10] Avvakumov GV, Walker JR, Xue S, Li Y, Duan S, Bronner C (2008). Structural basis for recognition of hemi-methylated DNA by the SRA domain of human UHRF1. Nature.

[11] Hashimoto H, Horton JR, Zhang X, Bostick M, Jacobsen SE, Cheng X (2008). The SRA domain of UHRF1 flips 5-methylcytosine out of the DNA helix. Nature.

[12] Babbio F, Pistore C, Curti L, Castiglioni I, Kunderfranco P, Brino L (2012). The SRA protein UHRF1 promotes epigenetic crosstalks and is involved in prostate cancer progression. Oncogene.

[13] Kim JK, Esteve PO, Jacobsen SE, Pradhan S (2009). UHRF1 binds G9a and participates in p21 transcriptional regulation in mammalian cells. Nucleic Acids Res.

[14] Mudbhary R, Hoshida Y, Chernyavskaya Y, Jacob V, Villanueva A, Fiel MI (2014). UHRF1 overexpression drives DNA hypomethylation and hepatocellular carcinoma. Cancer Cell.

[15] Kong X, Chen J, Xie W, Brown SM, Cai Y, Wu K (2019). Defining UHRF1 Domains that Support Maintenance of Human Colon Cancer DNA Methylation and Oncogenic Properties. Cancer Cell.

[16] Niinuma T, Kitajima H, Kai M, Yamamoto E, Yorozu A, Ishiguro K (2019). UHRF1 depletion and HDAC inhibition reactivate epigenetically silenced genes in colorectal cancer cells. Clin Epigenetics.

[17] Joseph JD, Darimont B, Zhou W, Arrazate A, Young A, Ingalla E (2016). The selective estrogen receptor downregulator GDC-0810 is efficacious in diverse models of ER+ breast cancer. eLife.

[18] Yoshida A, Tsuta K, Wakai S, Arai Y, Asamura H, Shibata T (2014). Immunohistochemical detection of ROS1 is useful for identifying ROS1 rearrangements in lung cancers. Modern pathology: an official journal of the United States and Canadian Academy of Pathology, Inc.

[19] Andersen JN, Sathyanarayanan S, Di Bacco A, Chi A, Zhang T, Chen AH (2010). Pathway-based identification of biomarkers for targeted therapeutics: personalized oncology with PI3K pathway inhibitors. Science translational medicine.

[20] Fang M, Li Y, Huang K, Qi S, Zhang J, Zgodzinski W (2017). IL33 Promotes Colon Cancer Cell Stemness via JNK Activation and Macrophage Recruitment. Cancer research.

[21] Sharif J, Muto M, Takebayashi S-i, Suetake I, Iwamatsu A, Endo TA (2007). The SRA protein Np95 mediates epigenetic inheritance by recruiting Dnmt1 to methylated DNA. Nature.

[22] Tian Y, Paramasivam M, Ghosal G, Chen D, Shen X, Huang Y (2015). UHRF1 contributes to DNA damage repair as a lesion recognition factor and nuclease scaffold. Cell reports.

[23] Li W, Sun W, Liu L, Yang F, Li Y, Chen Y (2010). IL-32: a host proinflammatory factor against influenza viral replication is upregulated by aberrant epigenetic modifications during influenza A virus infection. J Immunol.

[24] Li Y, Xie J, Xu X, Liu L, Wan Y, Liu Y (2013). Inducible interleukin 32 (IL-32) exerts extensive antiviral function via selective stimulation of interferon lambda1 (IFN-lambda1). J Biol Chem.

[25] Toffanin S, Hoshida Y, Lachenmayer A, Villanueva A, Cabellos L, Minguez B (2011). MicroRNA-based classification of hepatocellular carcinoma and oncogenic role of miR-517a. Gastroenterology.

[26] Li W, Xie L, He X, Li J, Tu K, Wei L (2008). Diagnostic and prognostic implications of microRNAs in human hepatocellular carcinoma. International journal of cancer.

[27] Unoki M, Nishidate T, Nakamura Y (2004). ICBP90, an E2F-1 target, recruits HDAC1 and binds to methyl-CpG through its SRA domain. Oncogene.

[28] Wurmbach E, Chen YB, Khitrov G, Zhang W, Roayaie S, Schwartz M (2007). Genome-wide molecular profiles of HCV-induced dysplasia and hepatocellular carcinoma. Hepatology.

[29] McConnell BB, Yang VW (2010). Mammalian Kruppel-like factors in health and diseases. Physiological reviews.

[30] Achour M, Jacq X, Ronde P, Alhosin M, Charlot C, Chataigneau T (2008). The interaction of the SRA domain of ICBP90 with a novel domain of DNMT1 is involved in the regulation of VEGF gene expression. Oncogene.

[31] Xie S, Jakoncic J, Qian C (2012). UHRF1 double tudor domain and the adjacent PHD finger act together to recognize K9me3-containing histone H3 tail. Journal of molecular biology.

[32] Gelato KA, Tauber M, Ong MS, Winter S, Hiragami-Hamada K, Sindlinger J (2014). Accessibility of different histone H3-binding domains of UHRF1 is allosterically regulated by phosphatidylinositol 5-phosphate. Molecular cell.

[33] Rothbart SB, Krajewski K, Nady N, Tempel W, Xue S, Badeaux AI (2012). Association of UHRF1 with methylated H3K9 directs the maintenance of DNA methylation. Nature structural & molecular biology.

[34] Ghiassi-Nejad Z, Hernandez-Gea V, Woodrell C, Lang UE, Dumic K, Kwong A (2013). Reduced hepatic stellate cell expression of Kruppel-like factor 6 tumor suppressor isoforms amplifies fibrosis during acute and chronic rodent liver injury. Hepatology.

[35] Solinas G, Schiarea S, Liguori M, Fabbri M, Pesce S, Zammataro L (2010). Tumor-conditioned macrophages secrete migration-stimulating factor: a new marker for M2-polarization, influencing tumor cell motility. Journal of immunology.

[36] Zhu XD, Zhang JB, Zhuang PY, Zhu HG, Zhang W, Xiong YQ (2008). High expression of macrophage colony-stimulating factor in peritumoral liver tissue is associated with poor survival after curative resection of hepatocellular carcinoma. Journal of clinical oncology: official journal of the American Society of Clinical Oncology.

[37] Ruffell B, Coussens LM (2015). Macrophages and Therapeutic Resistance in Cancer. Cancer Cell.

[38] Pollard JW (2009). Trophic macrophages in development and disease. Nature reviews Immunology.

[39] Martin-Sanz P, Casado M, Bosca L (2017). Cyclooxygenase 2 in liver dysfunction and carcinogenesis: Facts and perspectives. World J Gastroenterol.

[40] Leng J, Han C, Demetris AJ, Michalopoulos GK, Wu T (2003). Cyclooxygenase-2 promotes hepatocellular carcinoma cell growth through Akt activation: evidence for Akt inhibition in celecoxib-induced apoptosis. Hepatology.

[41] Xu L, Han C, Lim K, Wu T (2006). Cross-talk between peroxisome proliferator-activated receptor delta and cytosolic phospholipase A(2)alpha/cyclooxygenase-2/prostaglandin E(2) signaling pathways in human hepatocellular carcinoma cells. Cancer Res.

[42] Mayoral R, Fernandez-Martinez A, Bosca L, Martin-Sanz P (2005). Prostaglandin E2 promotes migration and adhesion in hepatocellular carcinoma cells. Carcinogenesis.

[43] Bottcher JP, Bonavita E, Chakravarty P, Blees H, Cabeza-Cabrerizo M, Sammicheli S (2018). NK Cells Stimulate Recruitment of cDC1 into the Tumor Microenvironment Promoting Cancer Immune Control. Cell.

[44] Dong ZR, Dong XF, Liu TQ, Zhi XT, Zou J, Zhong JT (2018). COX-2/PGE2 Axis Regulates HIF-2alpha Activity to Promote Hepatocellular Carcinoma Hypoxic Response and Reduce the Sensitivity of Sorafenib Treatment. Clin Cancer Res.

[45] Li Y, Fang M, Zhang J, Wang J, Song Y, Shi J (2016). Hydrogel dual delivered celecoxib and anti-PD-1 synergistically improve antitumor immunity. Oncoimmunology.

[46] Hmadcha A, Bedoya FJ, Sobrino F, Pintado E (1999). Methylation-dependent gene silencing induced by interleukin 1beta via nitric oxide production. J Exp Med.

[47] Peng D, Kryczek I, Nagarsheth N, Zhao L, Wei S, Wang W (2015). Epigenetic silencing of TH1-type chemokines shapes tumour immunity and immunotherapy. Nature.

[48] Arima Y, Hirota T, Bronner C, Mousli M, Fujiwara T, Niwa S (2004). Down-regulation of nuclear protein ICBP90 by p53/p21Cip1/WAF1-dependent DNA-damage checkpoint signals contributes to cell cycle arrest at G1/S transition. Genes Cells.

[49] Tien AL, Senbanerjee S, Kulkarni A, Mudbhary R, Goudreau B, Ganesan S (2011). UHRF1 depletion causes a G2/M arrest, activation of DNA damage response and apoptosis. Biochem J.

[50] Du S, Xu G, Zou W, Xiang T, Luo Z (2017). Effect of dihydroartemisinin on UHRF1 gene expression in human prostate cancer PC-3 cells. Anticancer Drugs.

[51] Seo JS, Choi YH, Moon JW, Kim HS, Park SH (2017). Hinokitiol induces DNA demethylation via DNMT1 and UHRF1 inhibition in colon cancer cells. BMC Cell Biol.

[52] Breinig M, Schirmacher P, Kern MA (2007). Cyclooxygenase-2 (COX-2)-a therapeutic target in liver cancer?. Curr Pharm Des.

[53] Chen H, Cai W, Chu ESH, Tang J, Wong CC, Wong SH (2017). Hepatic cyclooxygenase-2 overexpression induced spontaneous hepatocellular carcinoma formation in mice. Oncogene.

[54] Chen G, Li X, Yang J, Li J, Wang X, He J (2016). Prognostic significance of cyclooxygenase-2 expression in patients with hepatocellular carcinoma: a meta-analysis. Arch Med Sci.

[55] Booth L, Roberts JL, Cruickshanks N, Tavallai S, Webb T, Samuel P (2015). PDE5 inhibitors enhance celecoxib killing in multiple tumor types. J Cell Physiol.

[56] El-Kashef DH, El-Sheakh AR (2019). Hepatoprotective effect of celecoxib against tamoxifen-induced liver injury via inhibiting ASK-1/JNK pathway in female rats. Life Sci.

