# Supplementary material for: Crosstalk between macrophage-derived PGE2 and tumor UHRF1 drives hepatocellular carcinoma progression
Source: Theranostics. 2022 May 1;12(8):3776–93. doi: 10.7150/thno.69494 (PMC9131282; doi:10.7150/thno.69494)
Supplement: Supplementary file 1 — Supplementary figures and tables. [file thnov12p3776s1.pdf]

Supplementary Information

Crosstalk between macrophage-derived PGE_2 and tumor UHRF1 drives hepatocellular carcinoma progression

Jian Zhang[1,2#], Hongyan Zhang[1,2#], Xiuli Ding[1,2#], Jia Hu[2], Yongkui Li[2], Jinxiang Zhang[3], Hui Wang[4], Shanshan Qi[1,2], Aqing Xie[1,2], Jie Shi[2], Mengxi Xiang[1,2], Yawen Bin[2], Guobin Wang[5]*, Lin Wang[1,2]*, and Zheng Wang[2,5]*

[1] Department of Clinical Laboratory, Union Hospital, Tongji Medical College, Huazhong University of Science and Technology, Wuhan, Hubei, China 430022.

[2] Research Center for Tissue Engineering and Regenerative Medicine, Union Hospital, Tongji Medical College, Huazhong University of Science and Technology, Wuhan, Hubei, China 430022.

[3] Department of Surgery, Union Hospital, Tongji Medical College, Huazhong University of Science and Technology, Wuhan, Hubei, China 430022.

[4] Department of Human Genetics, Tongji Medical College, Huazhong University of Science and Technology, Wuhan, Hubei, China 430030.

[5] Department of Gastrointestinal Surgery, Union Hospital, Tongji Medical College, Huazhong University of Science and Technology, Wuhan, Hubei, China 430022

These authors contributed equally to this work.

Correspondence: Guobin Wang (wgb@hust.edu.cn), Lin Wang (lin_wang@hust.edu.cn), Zheng Wang (zhengwang@hust.edu.cn)

Table S1. Characteristics of HCC patients in Group 1.

**Variable**	
**Median age**	
< 50 years	26 (49.1%)
≥ 50 years	27 (50.9%)
**Pathological stage**	
I-II	39 (73.6%)
III-IV	14 (26.4%)
**AFP (ng/ml)**	
< 200	25 (47.2%)
≥ 200	28 (52.8%)
**Tumor size**	
< 5cm	14 (26.4%)
≥ 5cm	39 (73.6%)
**Tumor number**	
1	40 (75.5%)
≥ 2	13 (24.5%)

Values are shown as n (%). AFP, alpha fetoprotein.

Table S2. Sequences of primers.

qPCR primers	Sequences
GAPDH-S	AAGGCTGTGGGCAAGG
GAPDH-A	TGGAGGAGTGGGTGTCG
COX-2-S	CGGTGAAACTCTGGCTAGACAG
COX-2-A	GCAAACCGTAGATGCTCAGGGA
UHRF1-S	GACAAGCAGCTCATGTGCGATG
UHRF1-A	AGTACCACCTCGCTGGCATCAT
KLF6-S	CTGCCGTCTCTGGAGGAGT
KLF6-A	TCCACAGATCTTCCTGGCTGTC
CSF1-S	CCTGGGTCCTCTCGGC
CSF1-A	GGGAGCCCAGCCATGT
CSF2-S	AAACTTCCTGTGCAACCCAGA
CSF2-A	CCTTGGTCCCTCCAAGATGAC
CSF3-S	GCCCCAGGTAATTTCCTCCC
CSF3-A	GCCAAGACACTCACCCATCA
CXCL12-S	TGCCCTTCAGATTGTAGCCC
CXCL12-A	GCGTCTGACCCTCTCACATC
CCL14-S	GCATGAAGATCTCCGTGGCT
CCL14-A	CCCCCACCAACTTTAGCTGTAT
CCL18-S	GTTGACTATTCTGAAACCAGCCC
CCL18-A	GTCGCTGATGTATTTCTGGACCC
CCL13-S	GATCTCCTTGCAGAGGCTGAAG
CCL13-A	TCTGGACCCACTTCTCCTTTGG
U6-S	TTTGCGTGTCATCCTTGCG
U6-A	CTCGCTTCGGCAGCACAT
miR-520d-S	GAACATGTCTGCGTATCTC
miR-520d-A	CTACAAAGGGAAGCCCT
Uhrf1-S	AGTCTTCAGCCTCCGCACCTTT
Uhrf1-A	AGACCTCTCTGGCAACAAGCGT
Hprt-S	CCAGTTTCACTAATGACACAAAC
Hprt-A	CTGGTGAAAAGGACCTCTCGAAG
**ChIP primers**	
CSF1-CHIP-S	CATTTTCATCATCTAAGGGTCAG
CSF1-CHIP-A	TCCAAGCCTTCAGCAAACGAG
KLF6-CHIP-S	CCCGCACCATTGGCTCCA
KLF6-CHIP-A	AGCGGCGCAGAAGAGGACAG
miR-520d-CACCC-CHIP-S	CATGTACACACAAAAGACTC
miR-520d-CACCC-CHIP-A	GGGACAACTGTCCTGGGTTT
miR-520d-GGGCGG-CHIP-S	ACTTCAGCCTATGCGACA
miR-520d-GGGCGG-CHIP-A	CAAGACCAGCCTAACCAA
**Methyl-sequencing primers**	
CSF1 methyl-S	GAAGTTGGTTGGGTTGTTTGGGG
CSF1 methyl-A	CTATAATCATCCCAAAAAACTTT

Table S3. Primer sequences for shRNA and siRNAs.

shRNA

Gene name	Sequences (5' ->3')
UHRF1-1#-S	CCGGCGTCATTTACCACGTGAAATACTCGAGTATTTCACGTGGTAAATGACGTTTTTG
UHRF1-1#-A	AATTCAAAAACGTCATTTACCACGTGAAATACTCGAGTATTTCACGTGGTAAATGACG
UHRF1-2#-S	CCGGGCGCTGGCTCTCAACTGCTTTCTCGAGAAAGCAGTTGAGAGCCAGCGCTTTTTG
UHRF1-2#-A	AATTCAAAAAGCGCTGGCTCTCAACTGCTTTCTCGAGAAAGCAGTTGAGAGCCAGCGC
Uhrf1-1#-S	CCGGCTGTAGCTCCAGTGCCGTTAACTCGAGTTAACGGCACTGGAGCTACAGTTTTTG
Uhrf1-1#-A	AATTCAAAAACTGTAGCTCCAGTGCCGTTAACTCGAGTTAACGGCACTGGAGCTACAG
Uhrf1-2#-S	CCGGCACACACTCTTCGATTATGATCTCGAGATCATAATCGAAGAGTGTGTGTTTTTG
Uhrf1-2#-A	AATTCAAAAACACACACTCTTCGATTATGATCTCGAGATCATAATCGAAGAGTGTGTG
KLF6-1#-S	CCGGCCGTATGATGAGGCCAACTTTCTCGAGAAAGTTGGCCTCATCATACGGTTTTTG
KLF6-1#-A	AATTCAAAAACCGTATGATGAGGCCAACTTTCTCGAGAAAGTTGGCCTCATCATACGG
KLF6-2#-S	CCGGGATCCCATTGGTGAAGTCTTACTCGAGTAAGACTTCACCAATGGGATCTTTTTG
KLF6-2#-A	AATTCAAAAAGATCCCATTGGTGAAGTCTTACTCGAGTAAGACTTCACCAATGGGATC

siRNA

Gene name	Sequences (5' ->3')
CSF1-1#	GAUCCAGUGUGCUACCUUAAG
CSF1-2#	GGUCAAGAAUGUCUUUAAUGA
CCL14-1#	CCAUCGCCCUAGGGACCAATT
CCL14-2#	CCAACAGCCAGUGCUCCAATT
DNMT1-1#	GGUGUGCAUUGAUGCGGAATT
DNMT1-2#	GCACCUCAUUUGCCGAAUATT
G9a-1#	AAGCUCUAACUGAACAACUAA
G9a-2#	CACCAUGAACAUCGAUCGCAA

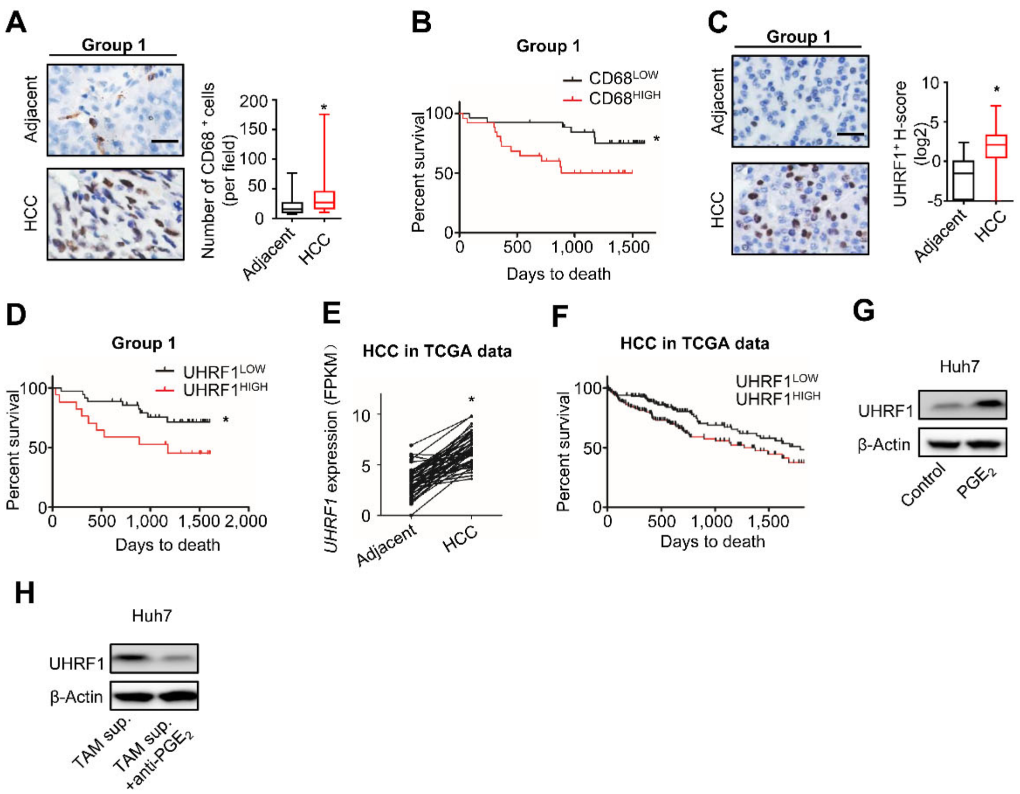

Figure S1. TAMs secrete PGE₂ to promote UHRF1 expression in HCC cells. (A) The representative images of CD68 expression (left panel) and numbers of $CD68^+$ cells (right panel) in human HCC samples (HCC) and their paired adjacent normal liver tissues (Adjacent) (Group 1). Scale bar, 50 μm. n = 53 patients**,** *P = 2 x 10^{-4}. Median, 25/75% quartiles (boxes), and Min.–Max. values (whisker) are shown. Student's *t*-test. **(B)** Kaplan-Meier overall survival stratified by $CD68^+$ TAMs in human HCC tissues (Group 1). n = 27 for $CD68^{LOW}$; n = 26 for $CD68^{HIGH}$. *P = 0.0217. Log-rank (Mantel-Cox) test. **(C)** The representative images of UHRF1 staining (left panel) and UHRF1 staining quantification (H-scores) (right panel) in HCC samples (HCC) and their paired adjacent normal liver tissues (Adjacent) (Group 1). Scale bar, 50 μm. n = 53, *P = 1.3 x 10^{-5}. Median, 25/75% quartiles (boxes), and Min.–Max. values (whisker) are shown. Student's *t*-test. **(D)** Kaplan-Meier overall survival stratified by UHRF1 expression levels in human HCC tissues (Group 1). n = 36 for $UHRF1^{LOW}$; n = 17 for $UHRF1^{HIGH}$. *P = 0.0430. Log-rank (Mantel-Cox) test. **(E)** *UHRF1* mRNA levels in human

HCC tissues and their paired adjacent normal liver tissues in The Cancer Genome Atlas (TCGA) database. n = 50, *P = 2.75 x 10^{-19}. Student's *t*-test. **(F)** Kaplan-Meier overall survival stratified by *UHRF1* mRNA levels in HCC patients in TCGA database. n = 149 for $UHRF1^{LOW}$; n = 181 for $UHRF1^{HIGH}$. *P = 0.0093. Log-rank (Mantel-Cox) test. **(G)** UHRF1 protein levels in Huh7 cells incubated with PGE_2 (200 ng/mL) for 24 hours. **(H)** UHRF1 protein levels in Huh7 cells incubated with human HCC TAM supernatants (TAM sup.) mixed without or with anti-PGE_2 (2 µg/mL) for 24 hours.

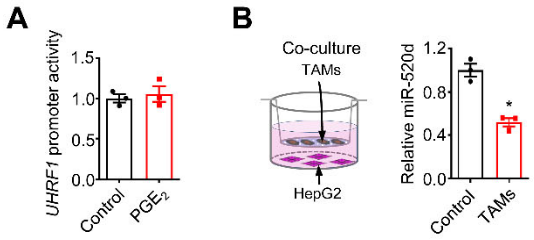

Figure S2. TAM-derived PGE_2 upregulates UHRF1 through inhibiting miR-520d. (A) The activity of luciferase driven by the *UHRF1* promoter in HepG2 cells in response to PGE_2 (200 ng/mL) treatment for 24 hours. **(B)** MiR-520d levels in HepG2 cells (lower chamber) cultured with medium (Control) or co-cultured with human HCC TAMs (TAMs) (upper chamber) for 24 hours. n = 3, *P = 0.0065. Student's *t*-test.

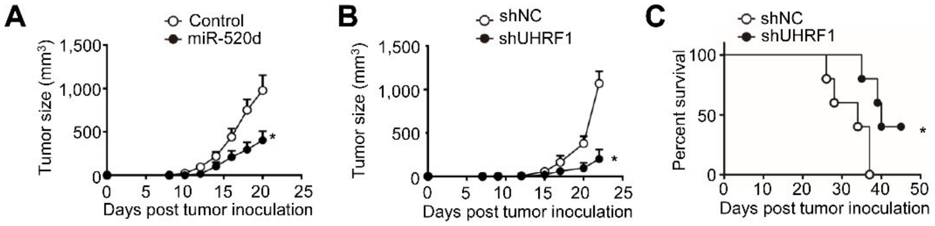

Figure S3. MiR-520d targets UHRF1 to control HCC progression. (A) Effect of miR-520d on tumor growth. 10^7 HepG2 cells stably expressing miR-520d or nonsense control miRNA (Control) were subcutaneously inoculated into nude mice. n = 8 per group, *P = 0.014. Student's *t*-test. **(B, C)** Effect of UHRF1 knockdown on tumor growth (**B**) and mouse survival (**C**). 10^7 HepG2 cells stably expressing shUHRF1 or nonsense control shRNA (shNC) were subcutaneously inoculated into nude mice. In **B**, n = 5 per group, *P = 0.00116, Student's *t*-test; in **C**, n = 5 per group, *P = 0.0163, Log-rank (Mantel-Cox) test.

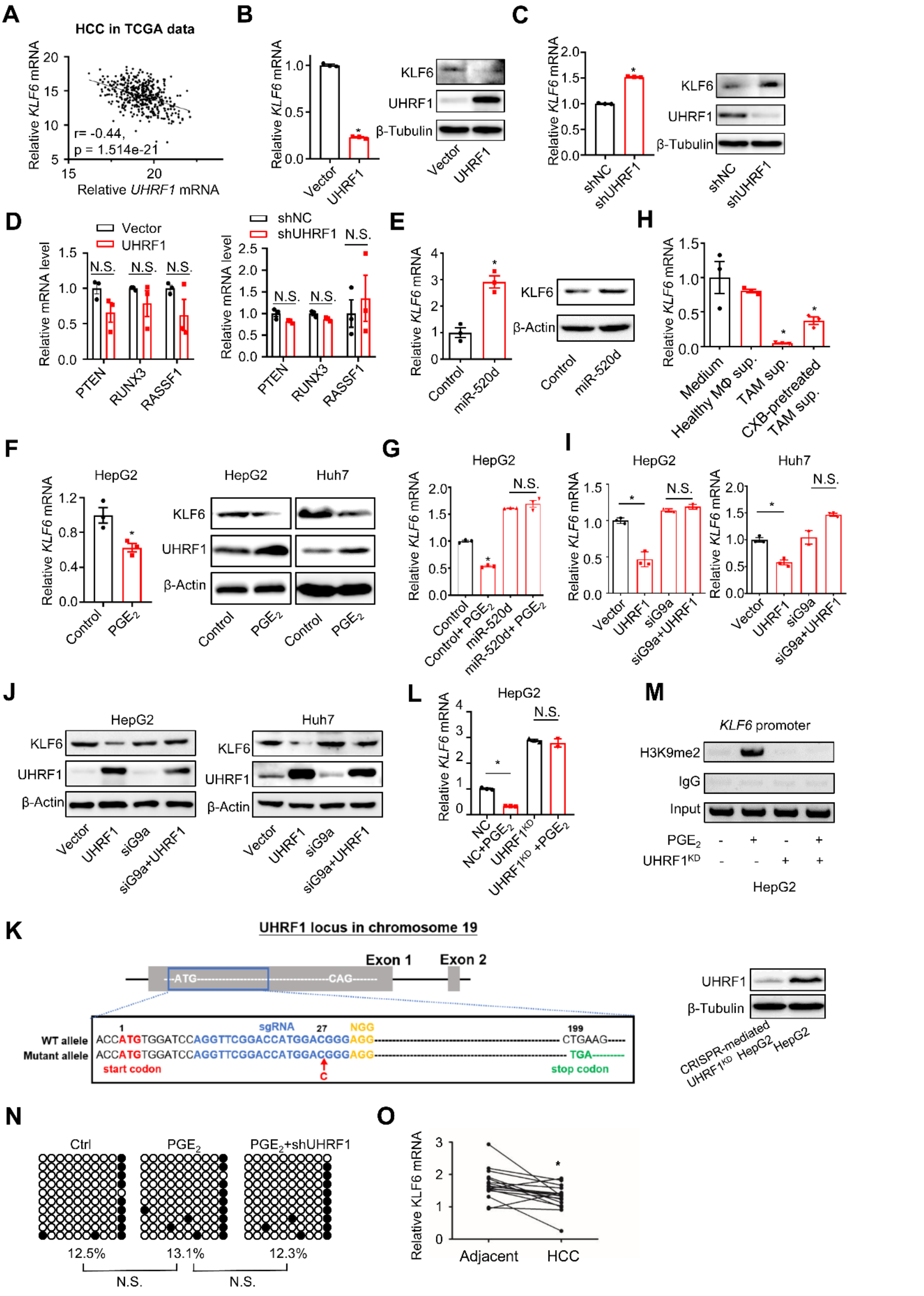

Figure S4. UHRF1 inhibits KLF6 through H3K9 methylation to promote HCC progression. (A) Correlation of *UHRF1* and *KLF6* in HCC. Mean log2 expression of *UHRF1* and *KLF6* in individual HCC tumors from TCGA RNA-seq data sets. The correlation

coefficient was shown. **(B)** KLF6 mRNA (left panel) and protein (right panel) levels in Huh7 cells overexpressing UHRF1 or control vector (Vector). n = 3, *P = 0.0043. Student's *t*-test. **(C)** KLF6 mRNA (left panel) and protein (right panel) levels in Huh7 cells overexpressing shUHRF1 or nonsense control shRNA (shNC). n = 3, *P = 0.0023. Student's *t*-test. **(D)** *PTEN*, *RUNX3* and *RASSF1* mRNA levels in HepG2 cells overexpressing UHRF1 (left) or shUHRF1 (right). n = 3. Student's *t*-test. **(E)** KLF6 mRNA (left panel) and protein (right panel) levels in HepG2 cells stably expressing nonsense control miRNA (Control) or miR-520d. n = 3, *P = 0.0028. Student's *t*-test. **(F)** KLF6 mRNA (left panel) and protein (middle and right panels) levels in HepG2 and Huh7 cells without or with PGE_2 (200 ng/mL) treatment for 24 hours. n = 3, *P = 0.0217. Student's *t*-test. **(G)** *KLF6* mRNA levels in HepG2 cells transfected with nonsense control miRNA (Control) or miR-520d mimics, followed by treating with PGE_2 (200 ng/mL) for 24 hours. **(H)** *KLF6* mRNA levels in HepG2 cells incubated with medium (Medium), supernatants of macrophages from healthy volunteers (Healthy MΦ sup.), supernatants of human HCC TAMs (TAM sup.), or supernatants of celecoxib-pretreated human HCC TAMs (CXB-pretreated TAM sup.) for 24 hours. n = 3, *P = 0.001101, TAM sup. Versus Medium; *P = 0.038, CXB-pretreated TAM sup. versus TAM sup. One-way ANOVA with Dunnett's multiple comparisons test. **(I, J)** Effect of overexpression UHRF1 and G9a knockdown (siG9a) in HepG2 and Huh7 cells on KLF6 mRNA (**I**) and protein (**J**) levels. Cells were transfected with UHRF1 expression plasmid and pooled siRNA against G9a for 24 hours. n = 3, *P < 0.05, versus corresponding control (Vector). Student's *t*-test. **(K)** Left: schematics showing genomic sequences of CRISPR/Cas9 edited *UHRF1* alleles in HepG2 cells. sgRNA target region (blue uppercases) was located in the first exon (gray box) of *UHRF1* gene. One *UHRF1* allele was wild-type (WT allele) and the other allele (Mutant allele) was mutated by the insertion of a base "C" (red arrow) immediately after Position 27 ("A" of ATG (red uppercases) as Position "1"), resulting in a frameshift inducing a premature stop codon (green uppercases) at Position 199, thus generating a UHRF1 knockdown genotype. Right: UHRF1 protein levels in HepG2 control

cells and CRISPR-mediated UHRF1-knockdown (UHRF1^{KD}) HepG2 cells. **(L)** *KLF6* mRNA levels in HepG2 control cells (NC) or CRISPR/Cas9-mediated UHRF1-knockdown (UHRF1^{KD}) HepG2 cells that were stimulated for 24 hours with 200 ng/ ml PGE_2. n = 3, *P < 0.05, Student's *t*-test. **(M)** ChIP assays showed that PGE_2 increased H3K9me2 in human *KLF6* promoter, which was abolished by CRISPR/Cas9-mediated UHRF1-knockdown (UHRF1^{KD}) in HepG2. **(N)** Bisulfite-sequencing analysis of the methylation of CpG islands at the promoter region of *KLF6* in HepG2 cells (Ctrl), PGE_2-treated HepG2 cells (PGE_2), and PGE_2-treated shUHRF1-expressing HepG2 cells. PGE_2, 200 ng/ ml. Filled circles, methylated; open circles, demethylated; below, percentages of methylated CpG to total CpG. **(O)** *KLF6* mRNA levels in primary HCC samples (HCC) and their paired adjacent normal liver tissues (Adjacent). 18 HCC patients, P = 0.03562. Student's *t*-test.

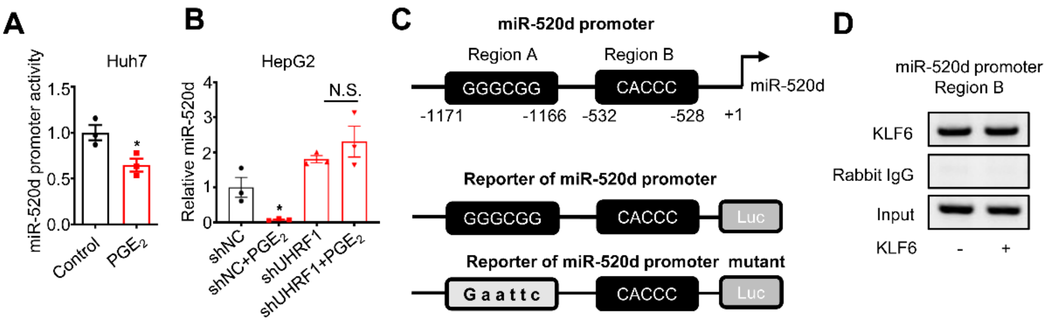

Figure S5. KLF6 and miR-520d form a molecular network in HCC. (A) The activity of luciferase driven by the miR-520d promoter in Huh7 cells treated with PGE_2 (200 ng/mL) for 24 hours. n = 3, *P = 0.0137. Student's *t*-test. **(B)** MiR-520d levels in shUHRF1 or shNC HepG2 cells treated with PGE2 (200 ng/mL) for 24 hours. **(C)** Schematic representation of the miR-520d promoter containing a "GC box" (Region A) and a CACCC element (Region B), and the luciferase (dark gray box) reporters driven by the miR-520d promoter and its "GC box" (Region A) mutants (lowercase; light gray box). **(D)** ChIP assays showing KLF6 occupancy on the Region B of the miR-520d promoter in HepG2 cells and KLF6-overexpressing HepG2 cells.

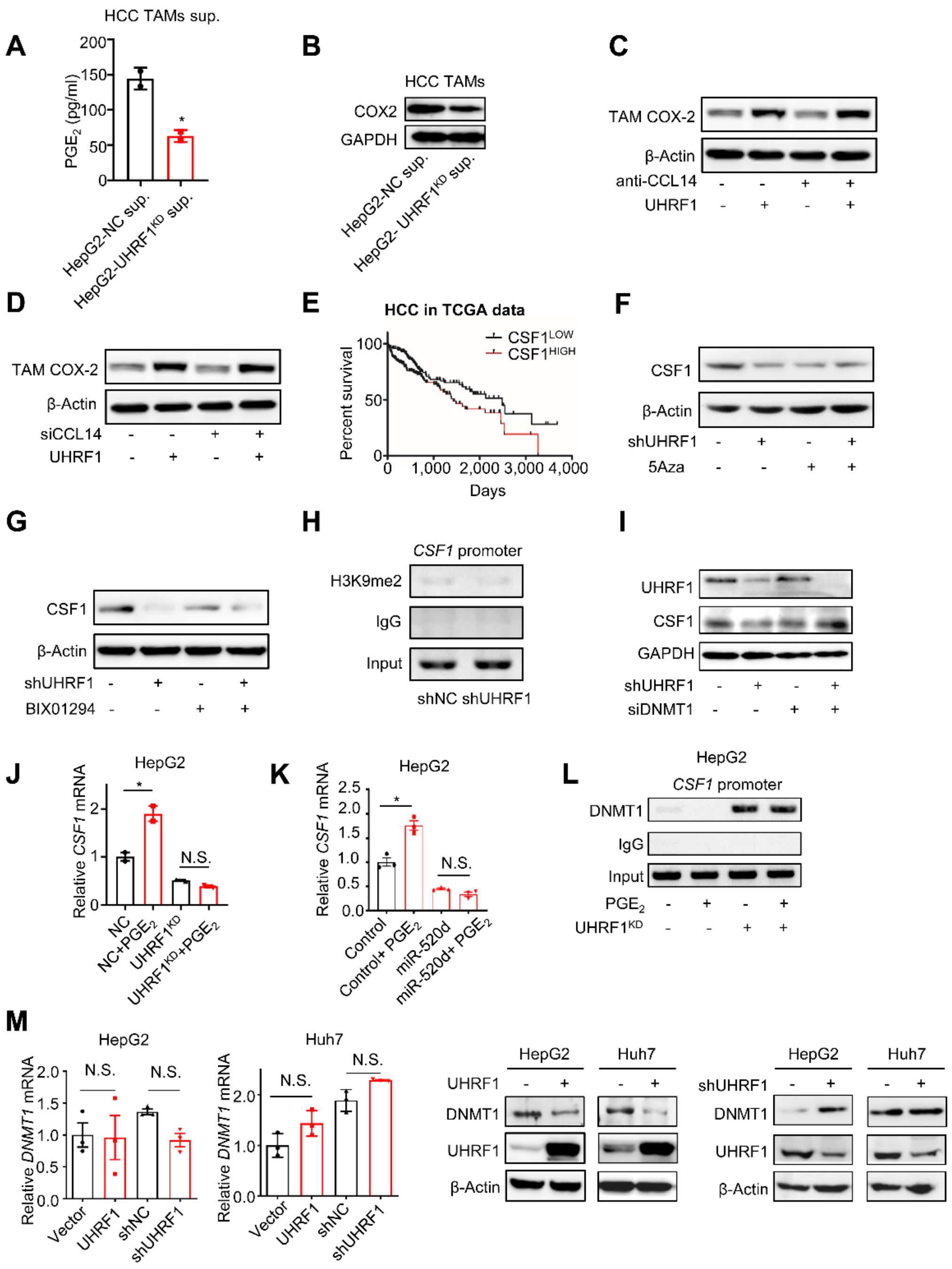

Figure S6. TAMs promote HCC progression via the UHRF1 and CSF1 network. (A) PGE_2 concentrations in the supernatants of human HCC TAMs that were cultured with the supernatants from HepG2 control cells (NC) or CRISPR/Cas9-mediated UHRF1-knockdown ($UHRF1^{KD}$) HepG2 cells for 24 hours followed by a replacement of fresh medium prior to measurement. n = 2. Student's *t*-test. **(B)** COX-2 protein levels in human HCC TAMs incubated with the supernatants from HepG2 control cells (NC) or CRISPR/Cas9-mediated UHRF1-knockdown ($UHRF1^{KD}$) HepG2 cells for 24 hours followed by a fresh medium replacement

prior to measurement. **(C)** COX-2 expression in human HCC TAMs incubated with the supernatants of HepG2 or UHRF1-overexpressing HepG2 that were treated without or with anti-CCL14 (2 μg/mL) for 24 hours. **(D)** COX-2 expression in human HCC TAMs incubated with the supernatants from HepG2 cells or UHRF1-overexpressing HepG2 that were treated without or with siRNA against CCL14 (siCCL14) for 48 hours. **(E)** Kaplan-Meier overall survival stratified by *CSF1* mRNA levels in HCC patients from TCGA database. n = 134 for $CSF1^{LOW}$; n = 196 for $CSF1^{HIGH}$. *P = 0.0151. Log-rank (Mantel-Cox) test. **(F)** CSF1 expression in shUHRF1-expressing HepG2 cells treated without or with 5-Aza (3 μM) for 24 hours. **(G)** CSF1 expression in shUHRF1-expressing HepG2 cells treated without or with BIX01294 for 24 hours. **(H)** ChIP assays showing H3K9me2 abundance in the *CSF1* promoter in HepG2 cells expressing nonsense control shRNA (shNC) or shUHRF1. **(I)** Effect of siRNA *DNMT1* on CSF1 protein levels in UHRF1-shRNA expressing Huh7 cells that were transfected with pooled siRNA against *DNMT1* for 24 hours. **(J)** *CSF1* mRNA levels in HepG2 control cells (NC) or CRISPR/Cas9-mediated UHRF1-knockdown ($UHRF1^{KD}$) HepG2 cells that were stimulated with 200 ng/ml PGE_2 for 24 hours. n = 3, *P < 0.05, Student's *t*-test. **(K)** *CSF1* mRNA levels in HepG2 cells transfected with nonsense control miRNA (Control) or miR-520d mimics, followed by treating with PGE_2 (200 ng/mL) for 24 hours. **(L)** ChIP assays showed that PGE_2 decreased DNMT1 association with *CSF1* promoter, which was abolished in CRISPR/Cas9-mediated UHRF1-knockdown ($UHRF1^{KD}$) HepG2 cells. **(M)** DNMT1 mRNA (left two panels) and protein (right two panels) levels in HepG2 and Huh7 cells overexpressing UHRF1 or shUHRF1. n = 3, P > 0.05, Student's *t*-test.
